# Dissecting causal relationships between gut microbiome, immune cells, and brain injury: A Mendelian randomization study

**DOI:** 10.1097/MD.0000000000039740

**Published:** 2024-09-20

**Authors:** Lina Xian, Xiaochen Xu, Yongmeng Mai, Tongwu Guo, Zhen Chen, Xiaoyan Deng

**Affiliations:** a Key Laboratory of Emergency and Trauma of Ministry of Education, Department of Intensive Care Unit, Key Laboratory of Hainan Trauma and Disaster Rescue, The First Affiliated Hospital, Hainan Medical University, Haikou, Hainan Province, PR China; b Department of Intensive Care Unit, Emergency and Trauma College, Hainan Medical University, The First Affiliated Hospital of Hainan Medical University, Haikou, Hainan Province, PR China; c Department of Intensive Care Unit, Shunde Hospital, Southern Medical University (the First people’s hospital of Shunde), Foshan, Guangdong Province, PR China.

**Keywords:** brain injury, gut microbiome, gut–brain axis, immune cells, mediation analyses, Mendelian randomization

## Abstract

Increasing literature has affirmed that changes in the gut microbiome (GM) composition were linked to distinct brain injury (BI) through the gut–brain axis, but it is uncertain if such links reflect causality. Further, the immune cell changes mediating the impact of GM on BI are not completely understood. We made use of the summary statistics of 211 GM (MiBioGen consortium), 731 immune cells, and 2 different BIs (FinnGen consortium), namely traumatic BI (TBI) and focal BI (FBI), from the extensive genome-wide association studies to date. We executed bidirectional Mendelian randomization (MR) analyses to ascertain the causal relationships between the GM and BI, and 2-step MR to validate possible mediating immune cells. Additionally, thorough sensitivity analyses verified the heterogeneity, robustness, as well as horizontal pleiotropy of the results. Based on the results of inverse-variance weighted (IVW) and sensitivity analyses, in MR analyses, 5 specific GM taxa and 6 specific GM taxa were causally associated with FBI and TBI, respectively; 27 immunophenotypes and 39 immunophenotypes were causally associated with FBI and TBI, respectively. Remarkably, *Anaerofilum*, *LachnospiraceaeNC2004group*, *RuminococcaceaeUCG004,* CCR2 on myeloid dendritic cell (DC), CD123 on CD62L+ plasmacytoid DC, and CD123 on plasmacytoid DC were causally associated with TBI and FBI (all *P* < .040). However, our reverse MR did not indicate any influence of TBI and FBI on the specific GM. In mediation analysis, we found that the associations between *Escherichia.Shigella* and FBI were mediated by CD123 on CD62L + plasmacytoid DC in addition to CD123 on plasmacytoid DC, each accounting for 4.21% and 4.21%; the association between *FamilyXIIIAD3011group* and TBI was mediated by CCR2 on myeloid DC, with mediated proportions of 5.07%. No remarkable horizontal pleiotropy or heterogeneity of instrumental variables was detected. Our comprehensive MR analysis first provides insight into potential causal links between several specific GM taxa with FBI/TBI. Additionally, CD123 on plasmacytoid DC in conjunction with CCR2 on myeloid DC may function in gut microbiota-host crosstalk in FBI and TBI, correspondingly. Further studies are critical to unravel the underlying mechanisms of the links between GM and BI.

## 1. Introduction

Brain injury (BI), including traumatic BI (TBI), ischemic and hemorrhagic stroke, anoxic BI, drug-related BI, and status epilepticus, all stand out as global health burdens, that culminate in lifelong disabilities or mortalities, resulting in a detrimental impact on injured people, their family and the whole society.^[1,2]^ Despite a segment of preclinical researchers who have made substantial strides in developing efficacious therapeutic approaches to avoid neuronal death and preserve neurological function, patients with BI have higher disabilities and poor prognosis.^[3]^ Additionally, treatment approaches to alleviate secondary BI following BI can culminate in deleterious off-target impacts on distant organ systems which may influence the the severity and prognosis of BI.^[4,5]^ Thus, further understanding the molecular physiology of disease and identifying novel effective treatment targets, which can prevent several BI concurrences or block secondary BI in survivors, is imperative.

Research delving into the function of the gut microbiome (GM) in regulating brain function, such as neurogenesis, neuroinflammation, and behavior, has exponentially heightened over the past decade, albeit predominantly in animal models.^[6,7]^ Pieces of literature have indulged in determining the bidirectional communication pathways between the microbiota of the central nervous system (CNS) and gut microbiota particularly recognized as the microbiota-gut–brain axis.^[8,9]^ Dysregulation of the aforementioned axis has been exceedingly linked to the pathophysiology of neurological disorders like Alzheimer disease,^[10]^ BI,^[11]^ Parkinson disease,^[12]^ stroke,^[13]^ and so on. For this reason, several researchers call the GM the “second brain.” Therefore, further comprehension of the effects of the microbiota-gut–brain axis in BI may introduce benefits clinically.

An observational study, recruiting 10 and 24 healthy control volunteers and TBI patients, correspondingly, investigated the characteristics of GM and stated that the abundance of *Enterococcus*, *Akkermansia*, *Parabacteroides*, and *Lachnoclostridium* was substantially increased, whereas the abundance of *Bifidobacterium* as well as *Faecalibacterium* were diminished among the TBI patients.^[14]^ Li *et al* found that ischemic stroke patients had significant GM dysbiosis with an increase in the abundance of short-chain fatty acids (SCFAs)-synthesizing bacteria, for instance, *Akkermansia* and *Odoribacter*, which was markedly linked to stroke outcome.^[15]^ Additionally, several animal’ studies ascertained that GM changes may control a pro-inflammatory response after BI and can worsen/relieve secondary BI. For example, Celorrio *et al*^[16]^ propounded that antibiotic-induced GM dysbiosis occurring prior to TBI remarkably exacerbated neuronal loss, minimized cortical infiltration of Ly6C^high^ monocytes in addition to T lymphocytes, enhanced microglial pro-inflammatory markers, and impeded neurogenesis following TBI. The above pieces of literature bolster the concept of modified GM composition playing a critical role in the development of BI. Nonetheless, the field is nascent, and data interpretation is frequently challenging provided that the microbiome composition is impacted by distinct factors encompassing exercise and diet.^[17,18]^ There is a certainty that these few observational studies were predisposed to reverse causation and confounding bias. It is important to ascertain if such links indicate spurious correlations or causal relations as a result of bias. Human randomized controlled trials (RCTs) are important in determining if targeting the specific GM can lead to the discovery of novel avenues for treatment.

In light of the lack of RCTs, Mendelian randomization (MR), conquers the bias because of 2 factors. These 2 factors are reverse causation and confounding. MR is a markedly acknowledged technique to assess more robust causal inferences between exposure and clinical outcomes by utilizing genetic variants like instrumental variables (IVs).^[19]^ Additionally, increasing literature illustrates the significance of utilizing human genetic data of GM features for clinical evaluations,^[20]^ which allowed us to deploy an MR approach to ascertain the mutually causal relations of BI and GM. In the present study, we execute a thorough MR analysis to ascertain the causal relationship between GM and BI for the first time. Additionally, animal and clinical studies have validated that GM may impact BI by controlling the immune cells, such as neutrophils,^[21]^ macrophages,^[16]^ and T cells.^[22]^ Thus, we projected that there might be causal relationships between immune cells, GM, and BI, then used mediation MR analysis to clarify the mediating function of these immune cells in the link between GM and BI.

## 2. Materials and methods

### 2.1. Study design overview

**Figure 1.**
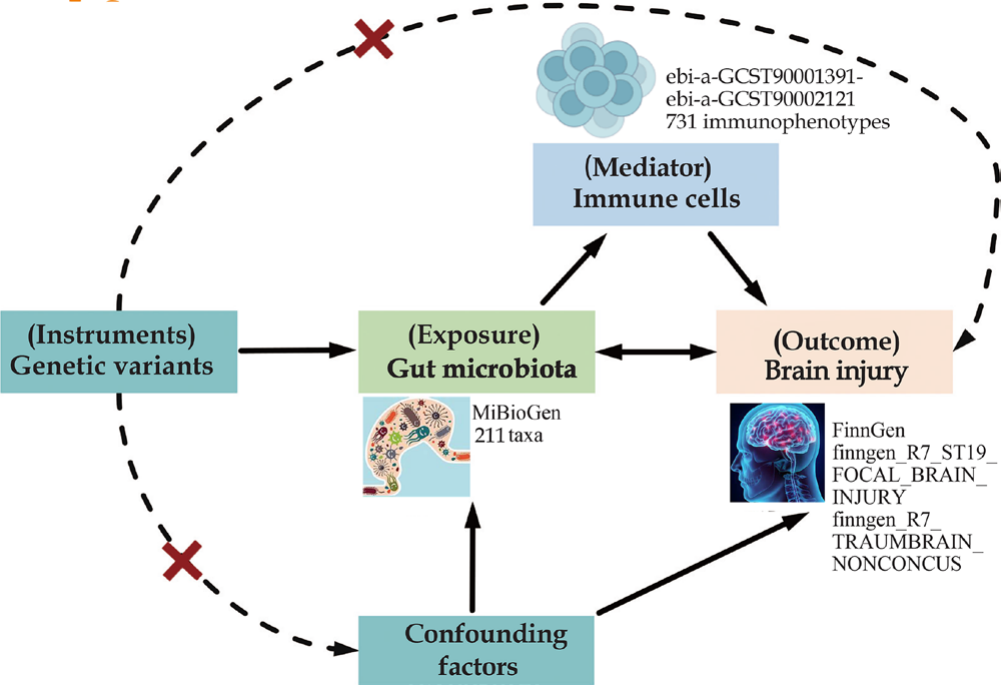
Assumptions and design of the bidirectional and mediation Mendelian randomization (MR) analyses. Firstly, a 2-sample bidirectional MR was performed to investigate the causal relationships between 211 gut microbiome (GM) (exposure) and brain injury (BI) (outcome). Secondly, 731 immune cells (mediator) were selected for subsequent mediation analyses. Finally, a 2-step MR analysis was conducted to detect potential mediating immunophenotypes (step 1, the effect of GM on immunophenotypes; step 2, the effect of immunophenotypes on immunophenotypes). The images for gut microbiota, immune cells, and BI were adapted from emojipng.com under the terms of the Non-Commercial Use License.

Our analytical strategy was as follows: first, we utilized a 2-sample bidirectional MR technique to examine the causal relationships among 211 GM and BI (2 subtypes) utilizing summary-level data gleaned from GWASs; second, we studied pieces of evidence on the causal relationships between 731 immune cells and BI (2 subtypes). Ultimately, we executed a mediation analysis to measure the percentage of the total effect of specific GM taxa on BI (2 subtypes) that was triggered by immune cells trait. Figure 1 exhibits a flow chart of the study design. The STROBE-MR (Strengthening the Reporting of Observational Studies in Epidemiology using Mendelian randomization) checklist was completed for this observational study. The original GWASs referred to herein have been ethically reviewed and approved by the relevant institutional review committee, therefore no additional ethics approval or participant consent is required for this research.

### 2.2. Data sources

#### 2.2.1. GWAS data sources for GM

Summary data of GM in the MiBioGen consortium (https://mibiogen.gcc.rug.nl) encompassed 18,340 participants of varied ancestries derived from 24 cohorts, predominantly of European descent.^[23]^ The MiBioGen consortium curated as well as evaluated the most comprehensive genome-wide meta-analysis amalgamating the 16S fecal microbiome and human genome-wide genotypes from participants. We only utilized the taxa that manifested in more than 10% of samples to detect genetic loci that influenced relative abundance (microbiome quantitative trait loci), leading to 211 taxa: 9 phyla, 16 classes, 20 orders, 35 families, and 131 genera. The GWAS data of all-inclusive cohorts were adjusted for variables, including age, sex, genetic main components, and other relevant factors.

#### 2.2.2. GWAS data sources for immune cells

GWAS summary statistics of immunological traits are available publicly from the GWAS Catalog (accession numbers GCST90001391 to GCST90002121).^[24]^ This dataset includes 731 distinct immune phenotypes, such as morphological parameters (MP) (n = 32), absolute cell (AC) counts (n = 118), relative cell (RC) counts (n = 192), and median fluorescence intensities (MFI) expressing surface antigen levels (n = 389) were incorporated. In particular, the MFI, AC, and RC features have B cells, myeloid cells, dendritic cells (DCs), monocytes, mature stages of T cells, TBNK (T cells, B cells, natural killer cells), and Treg panels, whereas the MP feature have TBNK and DC panels. The initial GWAS on immune traits was executed utilizing data from 3757 individuals of European origin and overlapping cohorts were absent. Nearly 22 million single-nucleotide polymorphisms (SNPs) genotyped that had high-density arrays were ascribed into the reference panel of the Sardinian sequence^[25]^ and associations were evaluated following adjusting for covariates (i.e., age, sex, and age^2^).

#### 2.2.3. GWAS data sources for BI

Large-scale GWAS data on FBI (GWAS ID: finn-b-ST19_FOCAL_BRAIN_INJURY) and TBI (GWAS ID: finn-b-TRAUMBRAIN_NONCONCUS) were obtained from the FinnGen Consortium. The FBI GWAS dataset was available from 137,641 European individuals (*N*_case_ = 1065, *N*_control_ = 136,576), with approximately 16.4 million variants examined postimputation and quality control. The TBI GWAS dataset was available from 218,792 European individuals (*N*_case_ = 3193, *N*_control_ = 215,599), with approximately 16.4 million variants examined after imputation and quality control.

### 2.3. Selection of IVs and data harmonization

The IVs in MR refer to genetic variants strongly associated with the exposure and not confounded by other factors that influence the outcome, which can adequately suppress the effect of confounding factors. There are 3 basic assumptions for a valid MR study that ought to be satisfied for the IVs (Fig. 1): (1) Relevance Assumption: SNPs markedly(*P* < 5 × 10^−8^ or *P* < 1 × 10^−5^, F-statistic > 10) linked to exposures are utilized as IVs; (2) Independence Assumption: SNPs (IVs) are not linked to pertinent confounding factors – that is, factors connected to both the exposure as well as the respective outcome; (3) Exclusivity Assumption: SNPs (IVs) influence outcome directly susceptibility via exposure and are not otherwise linked to outcome.

SNPs associated with GM and immunological traits were selected at the *P* < 1 × 10^−5^, as used in previous MR studies (given that only a small number of SNPs attained a degree of genome-wide significance (*P* < 5 × 10^−8^)).^[26,27]^ SNPs connected with BI were chosen at GWAS conventional thresholds (*P* < 5 × 10^−8^). Further, the examination of the linkage disequilibrium (LD) among SNPs relied on European ancestry reference data derived from the 1000 Genomes Project. We picked SNPs utilizing an LD coefficient (*r*^2^ < 0.001) and situated over 10Mb, to identify SNPs with independent genetic effects.

Additionally, in cases where there were no common SNPs between the outcome and exposure, proxies SNPs in LD (*r*^2^ ≥ 0.8) were added. To eliminate the impact of weak IV bias, we incorporated SNPs whose F-statistic was < 10 (a measurement of these IVs’ strength).

Noticeably, bidirectional MR evaluation between GM and BI was executed upon excluding IVs linked to immunological traits, MR analysis between immunological traits and BI was employed upon eliminating IVs linked to GM, MR evaluation between GM and immunological traits was employed upon eliminating IVs linked to BI.

### 2.4. Statistical analysis

The R version 4.3.1 of the package TwoSampleMR (version 0.5.6) executed the MR analyses. Unless a specified *P*-value is indicated, a 2-sided *P* < .05 connoted statistical significance.

#### 2.4.1. Primary MR analysis

MR analysis was employed to ascertain the causal relationships between GM and FBI to generate FBI-specific GM, between GM and TBI generate TBI-specific GM.

Regarding characteristics influenced by 2 or more SNPs, the random-effects inverse-variance weighted (IVW) models, which have stable, as well as, balanced pleiotropic impacts, were utilized as the primary approach to estimate causal effects. Effect estimates for trails controlled by a single SNP were derived utilizing the Wald ratio. The MR estimates are presented as odds ratio (OR) with a 95% CI for the dichotomous data or beta value with standard error (SE) for the continuous variables. To improve the reliability and strength of the causal relationship between specific GM and BI, we take the intersection of FBI-specific GM and TBI-specific GM, namely BI-specific GM.

#### 2.4.2. Reverse MR analysis

Using identical MR methods, we examined if FBI/TBI causally impacts GM traits and studied the possibility of reverse causation. FBI/TBI was taken into account as the exposure factor, and FBI-specific GM traits or TBI-specific GM were considered outcomes in this reverse MR analysis.

#### 2.4.3. MR mediation analysis

A stepwise MR evaluation technique identified the presence of mediation effects of immunological traits between FBI-specific GM traits and FBI, between TBI-specific GM traits and TBI. The 2-step MR presumed that no interaction between exposure and that of the mediator. The fundamental effect approximation of GM on BI (FBI and TBI) obtained from the univariate MR analyses, the total effect was defined as β1. Additionally, 2 other estimates need to be computed: (1) the causal exerted by the mediator (731 immune cells) on BI (FBI and TBI) was defined as β2, to improve the reliability and strength of the causal relationship between specific immunological traits and BI, we take the intersection of FBI-specific immune cells and TBI-specific immune cells, namely BI-specific immune cells; and (2) exposure causal effect (FBI-specific GM taxa or TBI-specific GM taxa) on the mediator (BI-specific immune cells) was denoted as β3. β2×β3 and β2×β3/β1 connoted the mediating and the percentage of the mediating effects, correspondingly.

#### 2.4.4. MR sensitivity and heterogeneity analysis

To ascertain if MR impact estimates are resilient to possible invalid genetic variants, we executed MR-Egger regression, weighted median (WM), simple mode, and weight mode as sensitivity analyses. In comparison to the IVW method, which presumes that all the SNPs are valid IVs when the Instrument Strength must be Independent of the Direct Effect (InSIDE) assumption is true, the MR-Egger regression test has the potential to produce a reliable estimate where every genetic instruments are deemed invalid. The WM model stands out as a robust method, capable of availing consistent estimate results when over half of the genetic instruments are deemed valid. For any possible heterogeneity, we applied Cochran Q statistic derived from the MR-Egger regression and IVW methods. They were visualized using funnel plots, while the MR-Egger intercept test tested horizontal pleiotropy, a threshold of *P* < .05 for both was utilized. Finally, we performed a leave-one-out (LOO) analysis to re-calibrate the overall effect size and explored whether the association can be affected by a single SNP, by eliminating 1 exposure-linked SNP at a time.

## 3. Results

### 3.1. Genetic instruments for GM and immune cells

By applying the indicated significance level (*P* < 1 × 10^−5^), LD clumping, harmonization, and F-statistics > 10, genetic IVs for 211 GM taxa and 731 immune cells were identified. Consequently, our study certainly exhibited negligible instrument bias.

### 3.2. Causal effects of GM on BI

**Figure 2.**
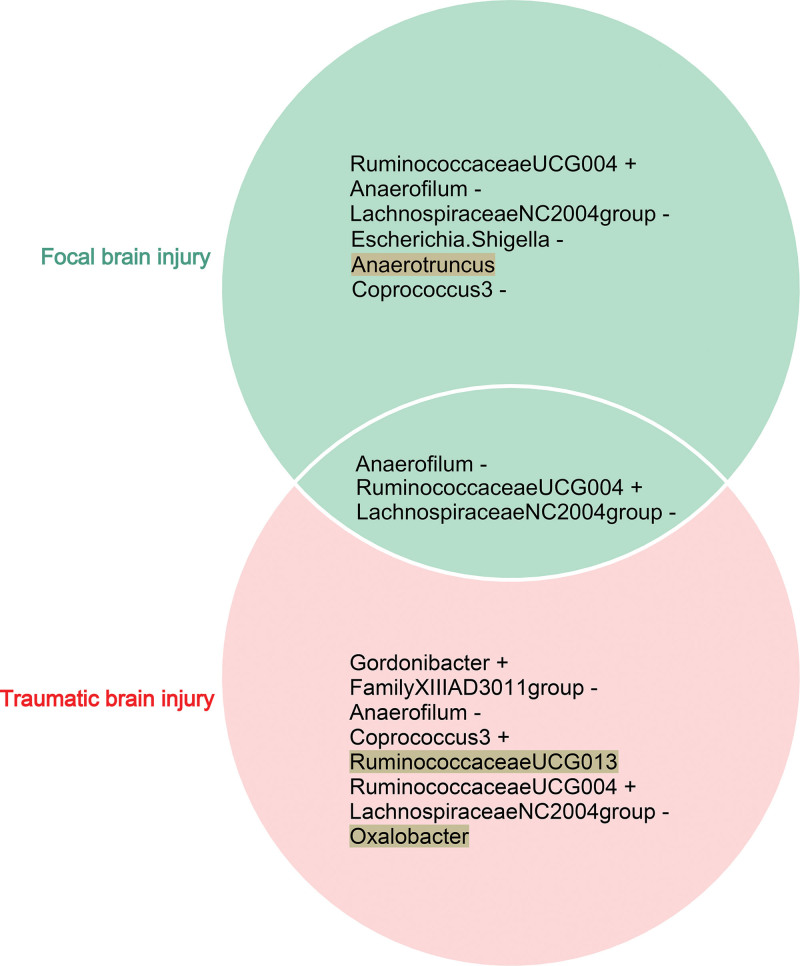
Venn diagram displays the shared GM in focal BI (FBI) and traumatic BI (TBI). + represents positive correlation, − represents negative correlation, text with color shading represent the result direction of inverse-variance weighted (IVW) method exists inconsistent with those of sensitivity analysis (MR-Egger regression, weighted median, simple model, and weight model).

**Figure 3.**
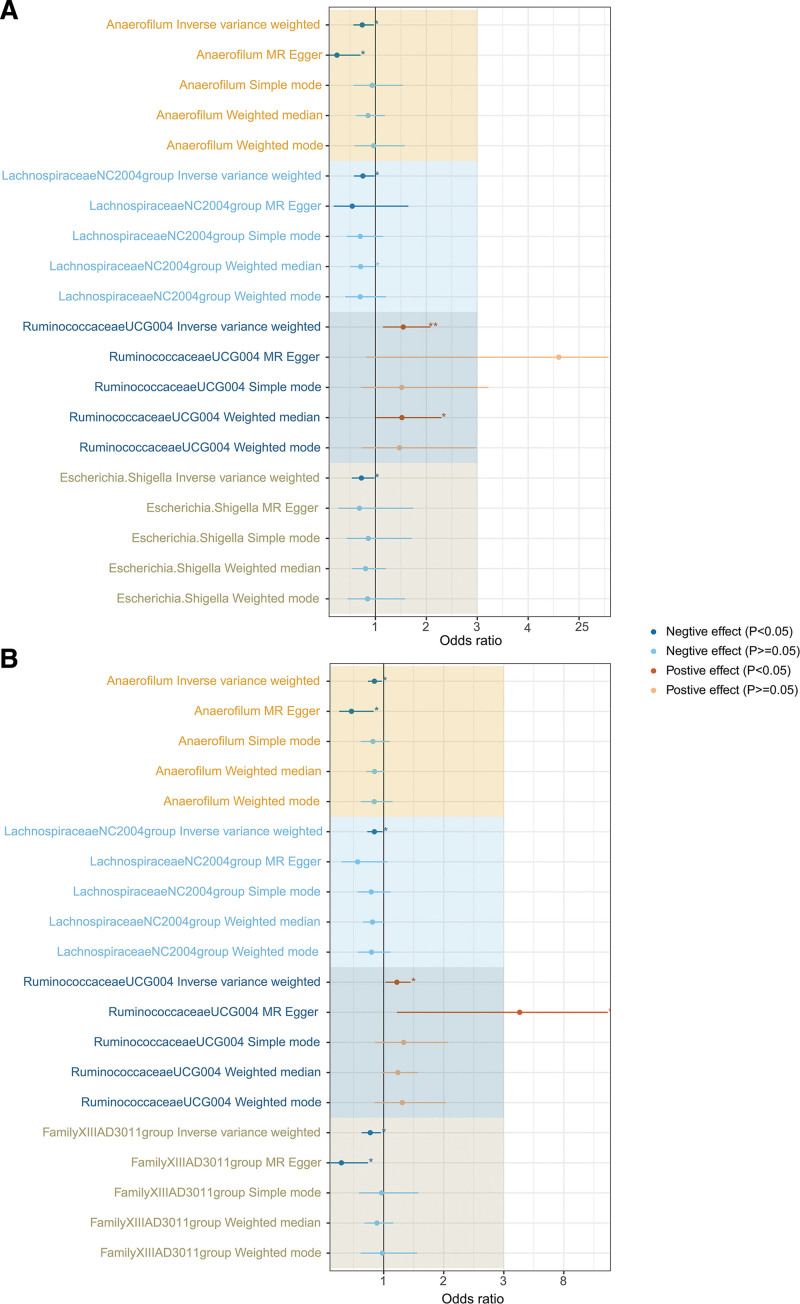
MR analysis evaluated the effects of GM on BI using the IVW method, MR-Egger regression, weighted median, simple model, and weight model (odds ratio with 95% confidence intervals). (A) Causal effect of genetically predicted GM on FBI. (B): Causal effect of genetically predicted GM on TBI. *P < .05; **P < .01; ***P < .001.

When examining the causal effects of GM on FBI, we screened 1423 SNPs as IVs from 211 GM taxa (Table S1, Supplemental Digital Content, http://links.lww.com/MD/N616). A total of 6 GM taxa (FBI-specific GM) yielded causal effects on FBI using the IVW method (*P* < .05) (Table S2, Supplemental Digital Content, http://links.lww.com/MD/N616 and Figure S1, Supplemental Digital Content, http://links.lww.com/MD/N615). Sensitivity analysis showed that only 5 GM taxa using the IVW method had the same direction as the findings of MR-Egger regression, WM, simple model, and weight model (Table S2, Supplemental Digital Content, http://links.lww.com/MD/N616 and Figure S2, Supplemental Digital Content, http://links.lww.com/MD/N615). When examining the causal effects of GM on TBI, we identified 1423 SNPs as IVs from 211 GM taxa (Table S3, Supplemental Digital Content, http://links.lww.com/MD/N616). A total of 8 GM taxa (TBI-specific GM) generated causal effects on TBI using the IVW method (*P* < .05) (Table S4, Supplemental Digital Content, http://links.lww.com/MD/N616 and Figure S3, Supplemental Digital Content, http://links.lww.com/MD/N615). Sensitivity analysis showed that only 6 GM taxa using the IVW method had the same direction as the findings of MR-Egger regression, WM, simple model, and weight model (Table S4, Supplemental Digital Content, http://links.lww.com/MD/N616 and Figure S4, Supplemental Digital Content, http://links.lww.com/MD/N615). To further improve the reliability and strength of the causal interrelationship between specific GM and BI, we take the intersection of FBI-specific GM and TBI-specific GM and consider the direction of action of the GM. As a result, 3 taxa (*Anaerofilum*, *LachnospiraceaeNC2004group,* and *RuminococcaceaeUCG004*) were causally associated with FBI and TBI (Fig. 2). In FBI, host-genetic-driven increase in *Anaerofilum* (OR = 0.74, 95% CI = 0.57–0.97, *P*_IVW_ = 0.028), *LachnospiraceaeNC2004group* (OR = 0.75, 95% CI = 0.58–0.98, *P*_IVW_ = 0.036), *Escherichia.Shigella* (OR = 0.72, 95% CI = 0.53–0.98, *P*_IVW_ = 0.037) exhibited significant protective effects on FBI, while genetically predicted higher relative abundances of *RuminococcaceaeUCG004* (OR = 1.55, 95% CI = 1.15–2.08, *P*_IVW_ = 0.004) (Table 1 and Fig. 3A) exhibited significant negative effects on FBI. In TBI, genetically predicted potentiates increase in *Anaerofilum* (OR = 0.84, 95% CI = 0.74–0.97, *P*_IVW_ = 0.016), *FamilyXIIIAD3011group* (OR = 0.78, 95% CI = 0.63–0.95, *P*_IVW_ = 0.016), *LachnospiraceaeNC2004group* (OR = 0.84, 95% CI = 0.73–0.98, *P*_IVW_ = 0.029) exhibited significant protective functions on TBI, while host-genetic-driven elevation in *RuminococcaceaeUCG004* (OR = 1.22, 95% CI = 1.03–1.45, *P*_IVW_ = 0.023) (Table 1 and Fig. 3B) exhibited significant negative effects on TBI.

**Table 1:** Mendelian randomization estimating the effect of gut microbiota exposure on the risk of brain injury.

Outcome	Exposure	Method	Number of SNP	Odds ratio (95% CI)	Beta ± SE	*P*	*Q*-statistics	*P*_h_	Egger intercept	*P*_intercept_
Focal brain injury	Anaerofilum	IVW	12	0.74 (0.57–0.97)	−0.299 ± 0.137	.028	16.14	.14		
		MR Egger	12	0.24 (0.08–0.71)	−1.411 ± 0.543	.027	11.20	.34	0.130	.06
		WM	12	0.86 (0.62–1.19)	−0.156 ± 0.166	.35				
	LachnospiraceaeNC2004group	IVW	10	0.75 (0.58–0.98)	−0.283 ± 0.135	.036	3.51	.94		
		MR Egger	10	0.54 (0.18–1.65)	−0.607 ± 0.565	.31	3.16	.92	0.037	.57
		WM	10	0.71 (0.51–0.99)	−0.344 ± 0.170	.043				
	RuminococcaceaeUCG004	IVW	12	1.55 (1.15–2.08)	0.436 ± 0.152	.004	8.84	.64		
		MR Egger	12	4.60 (0.82–25.72)	1.526 ± 0.878	.11	7.25	.70	−0.092	.24
		WM	12	1.52 (1.01–2.29)	0.419 ± 0.210	.046				
	Escherichia.Shigella	IVW	15	0.72 (0.53–0.98)	−0.323 ± 0.155	.037	10.50	.72		
		MR Egger	15	0.69 (0.27–1.74)	−0.377 ± 0.476	.44	10.49	.65	0.005	.91
		WM	15	0.81 (0.54–1.20)	−0.215 ± 0.204	.29				
Traumatic brain injury	Anaerofilum	IVW	12	0.84 (0.74–0.97)	−0.169 ± 0.070	.016	12.92	.30		
		MR Egger	12	0.46 (0.26–0.83)	−0770 ± 0.299	.029	8.66	.56	0.070	.07
		WM	12	0.85 (0.71–1.01)	−0.166 ± 0.089	.063				
	LachnospiraceaeNC2004group	IVW	10	0.84 (0.73–0.98)	−0.571 ± 0.323	.029	6.60	.68		
		MR Egger	10	0.57 (0.30–1.07)	−0.034 ± 0.015	.12	4.97	.71	0.047	.24
		WM	10	0.81 (0.66–1.00)	−0.035 ± 0.019	.052				
	RuminococcaceaeUCG004	IVW	12	1.22 (1.03–1.45)	0.198 ± 0.087	.023	10.50	.49		
		MR Egger	12	3.26 (1.22–8.73)	1.183 ± 0.502	.04	6.53	.77	−0.083	.07
		WM	12	1.24 (0.97–1.57)	0.211 ± 0.123	.085				
	FamilyXIIIAD3011group	IVW	15	0.78 (0.63–0.95)	−0.254 ± 0.105	.016	12.62	.25		
		MR Egger	15	0.30 (0.12–0.74)	−1.221 ± 0.467	.021	17.08	.48	0.078	.055
		WM	15	0.89 (0.68–1.16)	−0.120 ± 0.136	.38				

Odds ratios, 95% CI, and *P*-values were obtained from Mendelian randomization analysis. The heterogeneity test in the IVW and MR-Egger method was performed using Cochran *Q* statistic.

CI = confidence interval, IVW = inverse-variance-weighted_,_ MR = Mendelian randomization, *P*_h_ = *P*-value for heterogeneity, *P*_intercept_ = *P*-value for the intercept of the MR-Egger regression, SNP = single-nucleotide polymorphism, WM = weight median.

There was no obvious heterogeneity in the Cochran Q test utilizing MR-Egger regression and IVW (all *P* > .05, Table 1, Tables S5 and S6, Supplemental Digital Content, http://links.lww.com/MD/N616, and Figures S5 and S6, Supplemental Digital Content, http://links.lww.com/MD/N615). The MR-Egger regression intercepts failed to deviate from zero, a clear indication that horizontal pleiotropy was lacking (*P* > .05 for all intercepts, Table 1, Tables S7 and S8, Supplemental Digital Content, http://links.lww.com/MD/N616). Moreover, the LOO analyses ascertained that none of the detected causal interrelationships were driven by any individual IV (Figures S7 and S8, Supplemental Digital Content, http://links.lww.com/MD/N615).

### 3.3. Reverse MR analysis-causal effects of BI on GM

To mitigate the impact of reverse causality on the above findings, we executed a reverse MR analysis with FBI/TBI-specific GM traits as the outcome and FBI/TBI as the exposure variables. By utilizing the indicative significance level (*P* < 5 × 10^−8^), LD clumping, harmonization, and F-statistics > 10, a total of 13 and 103 SNPs were identified for FBI and TBI, respectively. MR analysis discovered no substantial reverse causal association of BI on FBI/TBI-specific GM was detected.

### 3.4. Causal functions of the mediator (immune cells) on BI

**Figure 4.**
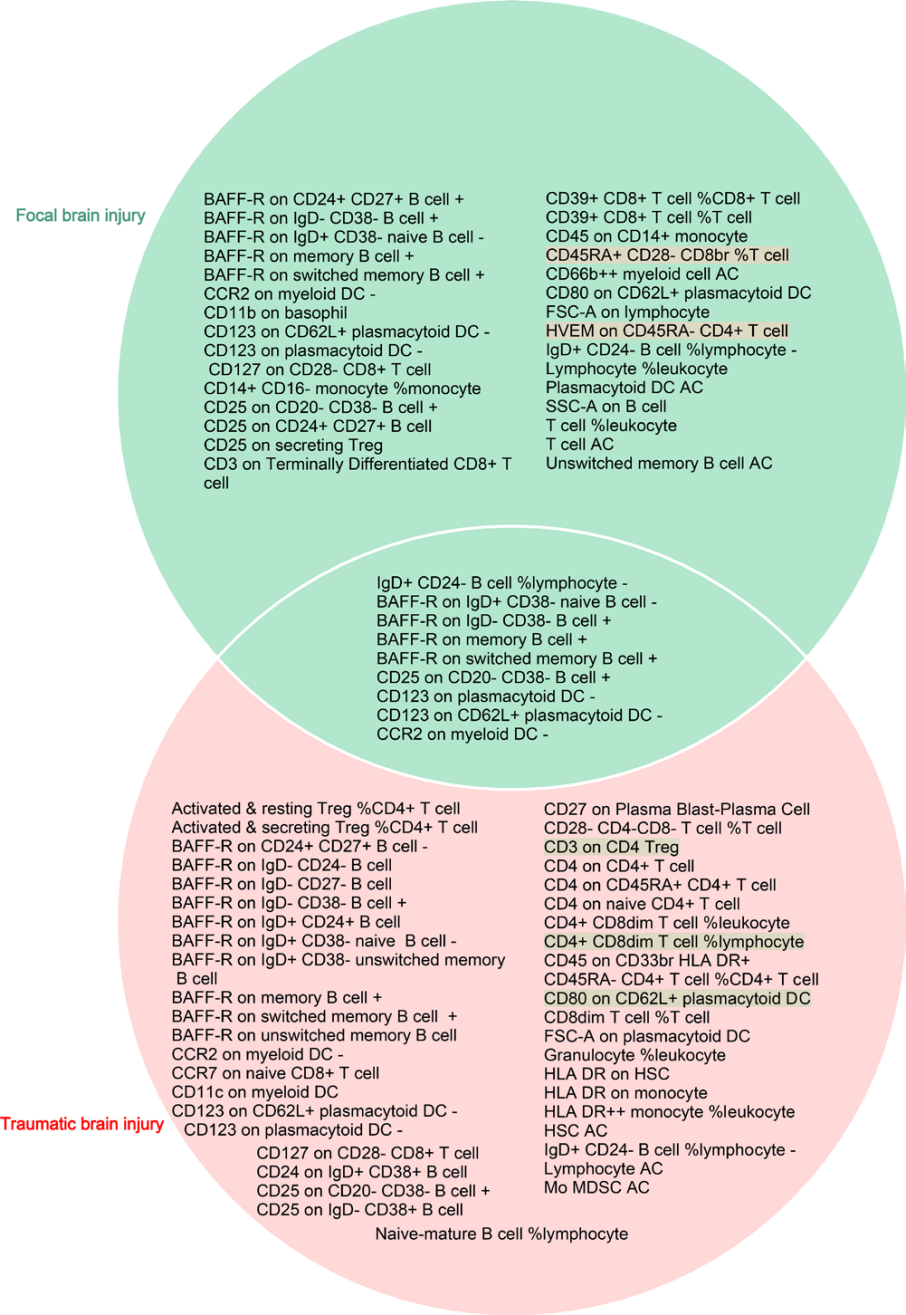
Venn diagram displays the shared immunophenotypes in focal BI (FBI) and traumatic BI (TBI). + represents positive correlation, − represents negative correlation, text with color shading represent the result direction of inverse-variance weighted (IVW) method exists inconsistent with those of sensitivity analysis (MR-Egger regression, weighted median, simple model, and weight model).

**Figure 5.**
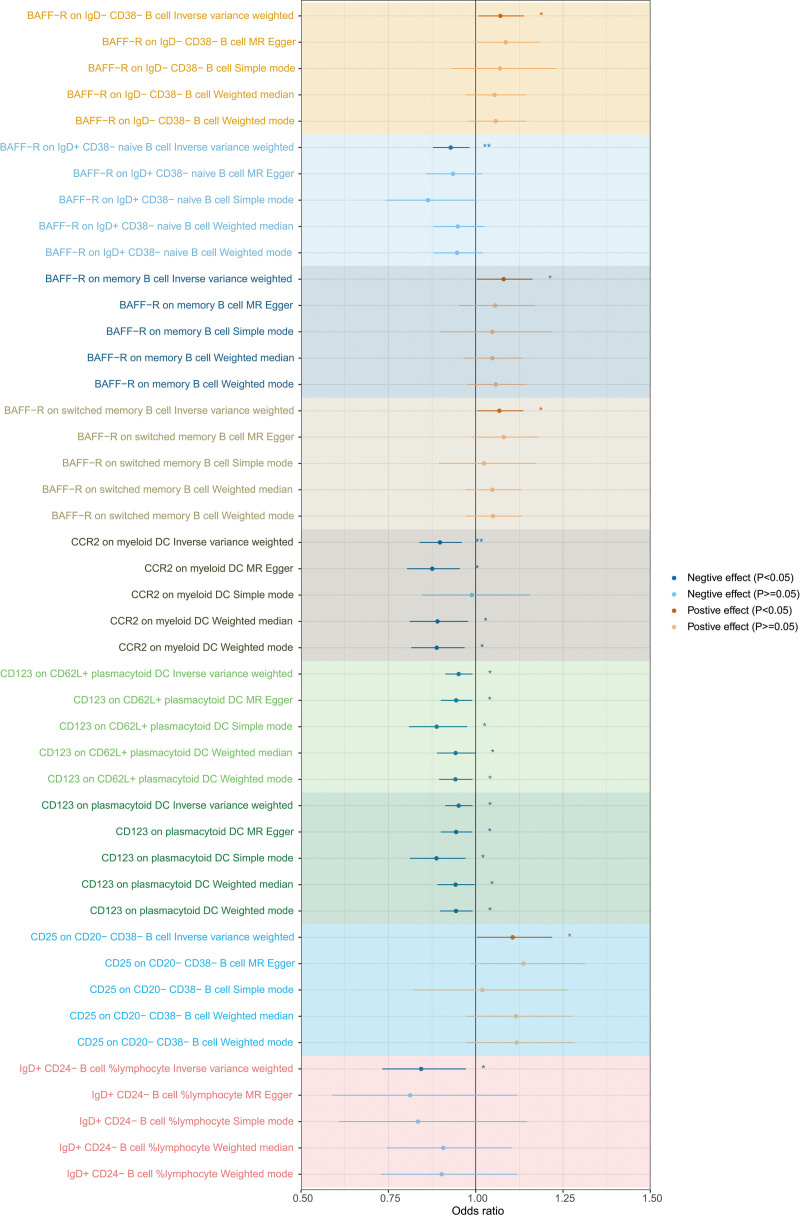
MR analysis evaluated the effects of shared immunophenotypes on FBI using the IVW method, MR-Egger regression, weighted median, simple model, and weight model (odds ratio with 95% confidence intervals). *P < .05; **P < .01; ***P < .001.

**Figure 6.**
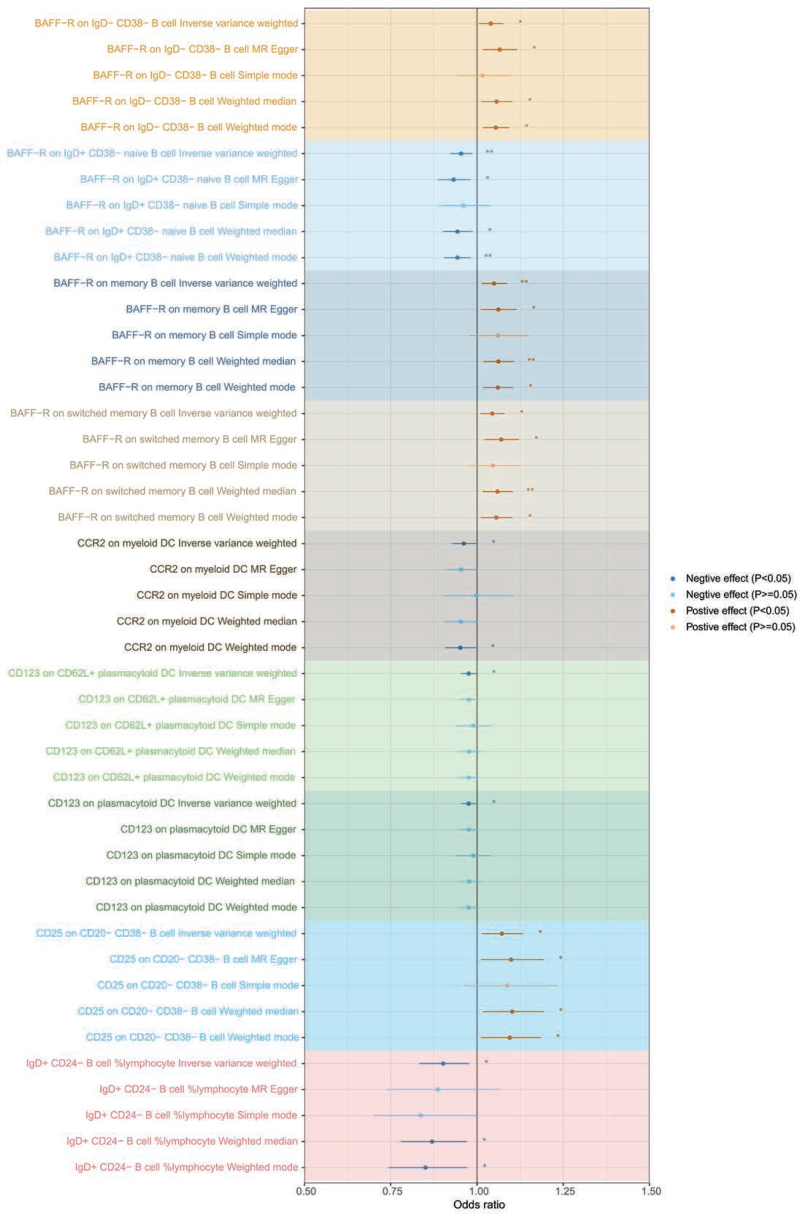
MR analysis evaluated the effects of shared immunophenotypes on TBI using the IVW method, MR-Egger regression, weighted median, simple model, and weight model (odds ratio with 95% confidence intervals). *P < .05; **P < .01; ***P < .001.

When evaluating the causal functions of immune cells on FBI, we screened 16,935 SNPs as IVs from 731 immune cell traits (Table S9, Supplemental Digital Content, http://links.lww.com/MD/N616). A total of 30 immunophenotypes (FBI-specific immune cells) yielded causal effects on FBI using the IVW method (*P* < .05) (Table S10, Supplemental Digital Content, http://links.lww.com/MD/N616 and Figure S9, Supplemental Digital Content, http://links.lww.com/MD/N615). Sensitivity analysis showed that only 27 immunophenotypes using the IVW method had the same direction as the findings of MR-Egger regression, WM, simple model, and weight model (Table S10, Supplemental Digital Content, http://links.lww.com/MD/N616 and Figure S10, Supplemental Digital Content, http://links.lww.com/MD/N615). When evaluating the causal effects of immune cells on TBI, we identified 16,935 SNPs as IVs from 731 immune cell traits (Table S11, Supplemental Digital Content, http://links.lww.com/MD/N616). A total of 42 immunophenotypes (TBI-specific immune cells) generated causal effects on TBI using the IVW method (*P* < .05) (Table S12, Supplemental Digital Content, http://links.lww.com/MD/N616 and Figure S11, Supplemental Digital Content, http://links.lww.com/MD/N615). Sensitivity analysis showed that only 39 immunophenotypes using the IVW method had the same direction as the findings of MR-Egger regression, WM, simple model, and weight model (Table S12, Supplemental Digital Content, http://links.lww.com/MD/N616 and Figure S12, Supplemental Digital Content, http://links.lww.com/MD/N615). To further improve the reliability and strength of the causal interrelationship between specific immune cells and BI, we take the intersection of FBI-specific immune cells and TBI-specific immune cells and consider the direction of action of the immune cells. As a result, 9 immune phenotypes (IgD + CD24- B cell %lymphocyte, BAFF-R on IgD + CD38- naive B cell, BAFF-R on IgD- CD38- B cell, BAFF-R on memory B cell, BAFF-R on switched memory B cell, CD25 on CD20- CD38- B cell, CD123 on plasmacytoid DC, CD123 on CD62L + plasmacytoid DC, and CCR2 on myeloid DC) were causally associated with FBI and TBI (Fig. 4). In FBI, host-genetic-driven increase in IgD + CD24- B cell %lymphocyte (OR = 0.84, 95% CI = 0.73–0.97, *P*_IVW_ = 0.019), BAFF-R on IgD + CD38- naive B cell (OR = 0.93, 95% CI = 0.88–0.98, *P*_IVW_ = 0.009), CD123 on plasmacytoid DC (OR = 0.95, 95% CI = 0.91–0.99, *P*_IVW_ = 0.021), CD123 on CD62L + plasmacytoid DC (OR = 0.95, 95% CI = 0.91–0.99, *P*_IVW_ = 0.016), and CCR2 on myeloid DC (OR = 0.90, 95% CI = 0.84–0.96, *P*_IVW_ = 0.002) exhibited significant protective effects on FBI, while genetically predicted higher relative abundances of BAFF-R on IgD- CD38- B cell (OR = 1.07, 95% CI = 1.01–1.14, *P*_IVW_ = 0.028), BAFF-R on memory B cell (OR = 1.08, 95% CI = 1.00–1.16, *P*_IVW_ = 0.041), BAFF-R on switched memory B cell (OR = 1.07, 95% CI = 1.00–1.14, *P*_IVW_ = 0.039), CD25 on CD20- CD38- B cell (OR = 1.11, 95% CI = 1.00–1.22, *P*_IVW_ = 0.043) (Table 2 and Fig. 5) exhibited significant negative effects on FBI. In TBI, genetically predicted increases increase in IgD + CD24- B cell %lymphocyte (OR = 0.90, 95% CI = 0.83–0.98, *P*_IVW_ = 0.012), BAFF-R on IgD + CD38- naive B cell (OR = 0.95, 95% CI = 0.92–0.99, *P*_IVW_ = 0.005), CD123 on plasmacytoid DC (OR = 0.98, 95% CI = 0.95–0.99, *P*_IVW_ = 0.039), CD123 on CD62L + plasmacytoid DC (OR = 0.98, 95% CI = 0.95–0.99, *P*_IVW_ = 0.039), and CCR2 on myeloid DC (OR = 0.96, 95% CI = 0.93–0.99, *P*_IVW_ = 0.040) exhibited significant protective effects on TBI, while host-genetic-driven increase in BAFF-R on IgD- CD38- B cell (OR = 1.04, 95% CI = 1.01–1.08, *P*_IVW_ = 0.024), BAFF-R on memory B cell (OR = 1.05, 95% CI = 1.01–1.09, *P*_IVW_ = 0.008), BAFF-R on switched memory B cell (OR = 1.04, 95% CI = 1.01–1.08, *P*_IVW_ = 0.012), CD25 on CD20- CD38- B cell (OR = 1.07, 95% CI = 1.01–1.13, *P*_IVW_ = 0.015) (Table 2 and Fig. 6) exhibited significant negative effects on TBI.

**Table 2:** Mendelian randomization estimating the effect of immune cells exposure on the risk of brain injury.

Outcome	Exposure	Method	Number of SNP	Odds ratio (95% CI)	Beta ± SE	*P*	*Q*-statistics	*P*_h_	Egger intercept	*P*_intercept_
Focal brain injury	BAFF-R on IgD− CD38− B cell	IVW	17	1.07 (1.01–1.14)	0.068 ± 0.031	.028	16.54	.42		
		MR Egger	17	1.09 (1.00–1.18)	0.082 ± 0.044	.079	16.30	.36	−0.011	.65
		WM	17	1.05 (0.97–1.14)	0.052 ± 0.042	.21				
	BAFF-R on IgD+ CD38− naive B cell	IVW	27	0.93 (0.88–0.98)	−0.074 ± 0.029	.009	19.51	.81		
		MR Egger	27	0.93 (0.86–1.02)	−0.067 ± 0.044	.14	19.47	.77	−0.004	.83
		WM	27	0.95 (0.88–1.02)	−0.052 ± 0.039	.18				
	BAFF-R on memory B cell	IVW	16	1.08 (1.00–1.16)	0.077 ± 0.038	.041	20.76	.14		
		MR Egger	16	1.06 (0.95–1.17)	0.054 ± 0.052	.32	20.17	.12	0.016	.53
		WM	16	1.05 (0.97–1.13)	0.046 ± 0.040	.25				
	BAFF-R on switched memory B cell	IVW	18	1.07 (1.00–1.14)	0.065 ± 0.032	.039	18.49	.36		
		MR Egger	18	1.08 (0.99–1.18)	0.077 ± 0.046	.11	18.33	.30	−0.009	.72
		WM	18	1.05 (0.97–1.13)	0.047 ± 0.039	.24				
	CCR2 on myeloid DC	IVW	14	0.90 (0.84–0.96)	−0.108 ± 0.034	.002	8.23	.83		
		MR Egger	14	0.88 (0.80–0.95)	−0.133 ± 0.044	.011	7.40	.83	0.017	.38
		WM	14	0.89 (0.81–0.98)	−0.116 ± 0.048	.016				
	CD123 on CD62L+ plasmacytoid DC	IVW	18	0.95 (0.91–0.99)	−0.050 ± 0.021	.016	14.16	.66		
		MR Egger	18	0.94 (0.90–0.99)	−0.058 ± 0.024	.031	13.80	.61	0.010	.55
		WM	18	0.94 (0.89–0.99)	−0.116 ± 0.048	.044				
	CD123 on plasmacytoid DC	IVW	18	0.95 (0.91–0.99)	−0.050 ± 0.021	.021	14.15	.66		
		MR Egger	18	0.88 (0.80–0.95)	−0.058 ± 0.024	.024	13.78	.61	0.010	.55
		WM	18	0.89 (0.81–0.98)	−0.060 ± 0.029	.029				
	CD25 on CD20− CD38− B cell	IVW	19	1.11 (1.00–1.22)	0.101 ± 0.050	.043	9.75	.94		
		MR Egger	19	1.14 (0.98–1.31)	0.128 ± 0.074	.11	9.49	.92	−0.010	.62
		WM	19	1.11 (0.97–1.28)	0.109 ± 0.069	.12				
	IgD+ CD24− B cell %lymphocyte	IVW	20	0.84 (0.73–0.97)	−0.170 ± 0.072	.019	22.38	.27		
		MR Egger	20	0.81 (0.58–1.12)	−0.208 ± 0.164	.22	22.29	.22	0.008	.80
		WM	20	0.91 (0.75–1.10)	−0.098 ± 0.100	.33				
**Traumatic brain injury**	BAFF-R on IgD− CD38− B cell	IVW	17	1.04 (1.01–1.08)	0.039 ± 0.017	.024	9.60	.89		
		MR Egger	17	1.07 (1.02–1.12)	0.063 ± 0.024	.017	7.31	.95	−0.019	.15
		WM	17	1.06 (1.01–1.10)	0.055 ± 0.022	.012				
	BAFF-R on IgD+ CD38− naive B cell	IVW	27	0.95 (0.92–0.99)	−0.047 ± 0.017	.005	27.85	.37		
		MR Egger	27	0.93 (0.89–0.98)	−0.070 ± 0.025	.011	26.38	.39	0.012	.25
		WM	27	0.94 (0.90–0.99)	−0.059 ± 0.023	.012				
	BAFF-R on memory B cell	IVW	16	1.05 (1.01–1.09)	0.048 ± 0.018	.008	14.79	.47		
		MR Egger	16	1.06 (1.01–1.11)	0.060 ± 0.025	.029	14.30	.43	−0.009	.49
		WM	16	1.06 (1.02–1.11)	0.060 ± 0.021	.005				
	BAFF-R on switched memory B cell	IVW	18	1.04 (1.01–1.08)	0.043 ± 0.017	.012	14.68	.62		
		MR Egger	18	1.07 (1.02–1.12)	0.068 ± 0.024	.012	12.50	.71	−0.019	.16
		WM	18	1.06 (1.02–1.10)	0.057 ± 0.021	.007				
	CCR2 on myeloid DC	IVW	14	0.96 (0.93–0.99)	−0.039 ± 0.019	.040	13.98	.38		
		MR Egger	14	0.95 (0.91–1.00)	−0.047 ± 0.024	.079	13.66	.32	0.006	.61
		WM	14	0.95 (0.91–1.00)	−0.048 ± 0.025	.055				
	CD123 on CD62L+ plasmacytoid DC	IVW	18	0.98 (0.95–0.99)	−0.025 ± 0.012	.039	13.92	.67		
		MR Egger	18	0.98 (0.95–1.00)	−0.024 ± 0.014	.10	13.91	.61	−0.001	.93
		WM	18	0.98 (0.94–1.01)	−0.023 ± 0.018	.21				
	CD123 on plasmacytoid DC	IVW	18	0.98 (0.95–0.99)	−0.025 ± 0.012	.039	13.91	.67		
		MR Egger	18	0.98 (0.95–1.00)	−0.024 ± 0.014	.10	13.90	.61	−0.001	.93
		WM	18	0.98 (0.94–1.01)	−0.023 ± 0.018	.21				
	CD25 on CD20− CD38− B cell	IVW	19	1.07 (1.01–1.13)	0.069 ± 0.028	.015	10.82	.90		
		MR Egger	19	1.10 (1.01–1.19)	0.094 ± 0.042	.040	10.21	.89	−0.009	.45
		WM	19	1.10 (1.02–1.19)	0.097 ± 0.041	.018				
	IgD+ CD24− B cell % lymphocyte	IVW	20	0.90 (0.83–0.98)	−0.103 ± 0.041	.012	21.89	.29		
		MR Egger	20	0.89 (0.74–1.06)	−0.120 ± 0.093	.21	21.84	.24	0.003	.84
		WM	20	0.87 (0.78–0.97)	−0.140 ± 0.056	.013				

Odds ratios, 95% CI, and *P*-values were obtained from Mendelian randomization analysis. The heterogeneity test in the IVW and MR-Egger method was performed using Cochran *Q* statistic.

CI = confidence interval, DC = dendritic cell, IVW = inverse-variance-weighted, MR = Mendelian randomization, *P*_h_ = *P*-value for heterogeneity, *P*_intercept_ = *P*-value for the intercept of the MR-Egger regression, SNP = single-nucleotide polymorphism, WM = weight median.

Obvious heterogeneity in the Cochran Q test was lacking when utilizing IVW and MR-Egger regression (all *P* > .05, Table 2, Tables S13 and S14, Supplemental Digital Content, http://links.lww.com/MD/N616 and Figure S13 and S14, Supplemental Digital Content, http://links.lww.com/MD/N615). The MR-Egger regression intercepts failed to deviate from zero, signifying no indication of horizontal pleiotropy (*P* > .05 for all intercepts, Table 2, Tables S15 and S16, Supplemental Digital Content, http://links.lww.com/MD/N616). Moreover, the LOO analyses affirmed that none of the detected causal interrelationships were driven by any individual IV (Figures S15 and S16, Supplemental Digital Content, http://links.lww.com/MD/N615).

### 3.5. Causal effects of the exposure (GM) on the mediator (immune cells)

After identifying the mediator (9 immunophenotypes), we investigate the causal associations of FBI/TBI-specific GM on it. As a result, *Escherichia.Shigella* was significantly positively associated with CD123 on CD62L + plasmacytoid DC (OR = 1.31, 95% CI = 1.02–1.69, *P*_IVW_ = 0.035), and CD123 on plasmacytoid DC (OR = 1.31, 95% CI = 1.02–1.69, *P*_IVW_ = 0.034) (Table 3). *FamilyXIIIAD3011group* was significantly positively correlated with CCR2 on myeloid DC (OR = 1.39, 95% CI = 1.05–1.84, *P*_IVW_ = 0.021) (Table 3). Importantly, the results of the other 4 analytical methods concurred with the IVW direction, unifying the cause-effect interrelationship (Table S17, Supplemental Digital Content, http://links.lww.com/MD/N616). No evidence of heterogeneity and horizontal pleiotropy (all *P* > .05) was observed among the IVs, denoting the reliability of the results (Table 3). The LOO analyses verified that no individual IV markedly influenced the detected causal associations.

**Table 3:** Mendelian randomization analyses of the causal effects between gut microbiota and immune cells.

Mediator and outcome	Exposure	Method	Number of SNP	Odds ratio (95% CI)	Beta ± SE	*P*	*Q*-statistics	*P*_h_	Egger intercept	*P*_intercept_
CD123 on CD62L+ plasmacytoid DC Focal brain injury	Escherichia.Shigella	IVW	15	1.31 (1.02–1.69)	0.271 ± 0.129	.035	15.26	.36		
		MR Egger	15	1.13 (0.53–2.41)	0.121 ± 0.387	.76	15.07	.30	0.013	.69
		WM	15	1.37 (0.98–1.91)	0.314 ± 0.170	.065				
CD123 on plasmacytoid DC Focal brain injury	Escherichia.Shigella	IVW	15	1.31 (1.02–1.69)	0.272 ± 0.128	.034	15.13	.37		
		MR Egger	15	1.13 (0.53–2.40)	0.121 ± 0.387	.76	14.93	.31	0.013	.68
		WM	15	1.37 (0.99–1.89)	0.315 ± 0.164	.054				
CCR2 on myeloid DC Traumatic brain injury	FamilyXIIIAD3011group	IVW	14	1.39 (1.05–1.84)	0.330 ± 0.143	.021	15.52	.28		
		MR Egger	14	1.58 (0.37–6.72)	0.450 ± 0.743	.56	15.49	.22	−0.010	.87
		WM	14	1.34 (0.95–1.91)	0.296 ± 0.179	.098				

Odds ratios, 95% CI, and *P*-values were obtained from Mendelian randomization analysis. The heterogeneity test in the IVW and MR-Egger method was performed using Cochran *Q* statistic.

CI = confidence interval, DC = dendritic cell, IVW = inverse-variance-weighted_,_ MR = Mendelian randomization, *P*_adj_ = *P*-value adjusted, *P*_h_ = *P*-value for heterogeneity, *P*_intercept_ = *P*-value for the intercept of the MR-Egger regression, SNP = single-nucleotide polymorphism, WM = weight median.

### 3.6. Mediating effects of immune cell traits on GM→BI effects

**Figure 7.**
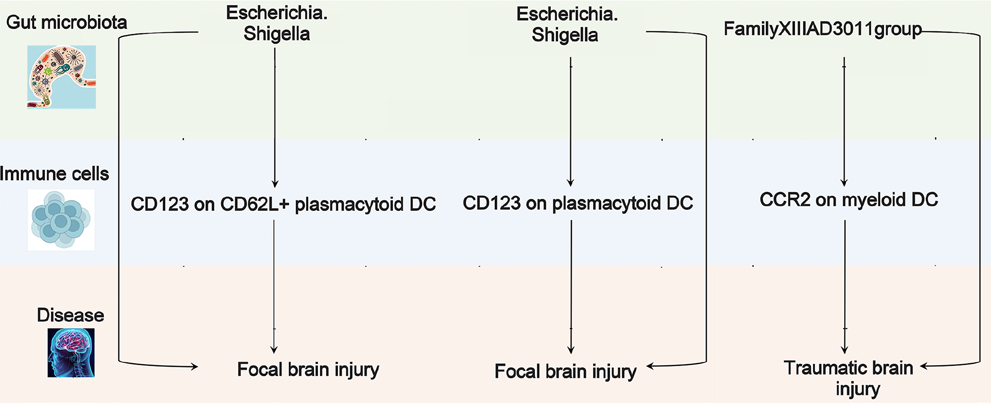
MR analyses show causal effects of immunophenotypes on GM and FBI/TBI. The diagram displays the mediation mode of “GM-immune cells-FBI/TBI” in 2-step MR. Beta values (β) indicate the causal effect estimates using the in IVW method (truncated at *P *< .05).

Based on “GM→FBI/TBI,” “immune cells→FBI/TBI” and“GM→immune cells” in the previous analysis, we considered immune cells to mediate the relationship between GM and FBI/TBI (Fig. 7). Specifically, CD123 on CD62L + plasmacytoid DC and CD123 on plasmacytoid DC mediated the effects of *Escherichia.Shigella* on FBI, with mediating impacts of −0.0136, and −0.0136, denoting 4.21%, and 4.21% of the total effects, correspondingly (Table 4). CCR2 on myeloid DC mediated the effects of *FamilyXIIIAD3011group* on TBI, with mediating effects of −0.0129, reflecting 5.07% of the total effects (Table 4).

**Table 4:** Mediation Mendelian randomization analysis of the causal effects between gut microbiota, immune cells, and brain injury.

Exposure	Mediator	Outcome	Total effect (β1)	A (β2)	B (β3)	Indirect effect (β)	Proportion mediated
Escherichia.Shigella	CD123 on CD62L+ plasmacytoid DC	Focal brain injury	−0.323	−0.050	0.271	−0.0136	4.21%
Escherichia.Shigella	CD123 on plasmacytoid DC	Focal brain injury	−0.323	−0.050	0.272	−0.0136	4.21%
FamilyXIIIAD3011group	CCR2 on myeloid DC	Traumatic brain injury	−0.254	−0.039	0.330	−0.0129	5.07%

A = the effect of mediation on outcome, B = the effect of exposure on mediation, DC = dendritic cell.

## 4. Discussion

The present study utilized large-scale GWAS summary data and a 2-sample MR technique to ascertain the relationship between 211 GM, 731 immune cell subtypes, and FBI/TBI. Our genetic analyses found several potential causal associations between GM and FBI/TBI, and further analysis revealed that immunophenotypes mediated a partial proportion of the causal effect of GM on the FBI/TBI risk. Our findings indicated that different GM taxa exert different effects on FBI/TBI, underlying the necessity of controlling host–microbe balance in the prevention and management of FBI/TBI.

Accumulating evidence supported an association between GM abundance and BI,^[14,15,28]^ however, there are deficient direct pieces of evidence affirming a causal correlation. Considering that BI comprises a heterogeneous group with diverse disease entities (such as TBI, and FBI), each accompanied by individual pathophysiology and variations. MR analysis investigated the causal relationship between 211 GM taxa and TBI and FBI, respectively. As a result, a total of 5 genetically predicted GM taxa (*RuminococcaceaeUCG004, Anaerofilum, LachnospiraceaeNC2004group, Escherichia.Shigella, Coprococcus3*) and 6 genetically predicted GM taxa (*RuminococcaceaeUCG004, Anaerofilum, LachnospiraceaeNC2004group, FamilyXIIIAD3011group, Coprococcus3, Gordonibacter*) significantly affected FBI and TBI, respectively, using IVW method had the same direction as the findings of MR-Egger regression, WM, simple model and weight model. It is worth noting that there are 3 shared GM taxa (*RuminococcaceaeUCG004, Anaerofilum, LachnospiraceaeNC2004group*) that have a significant causal relationship with FBI and TBI. Within the *Ruminococcaceae* family, *RuminococcaceaeUCG004* represents a genus of anaerobic bacteria. Urban et al^[29]^ employed a 2-site trial to ascertain the function of the gut–brain axis in comorbidities connected with chronic TBI and compared the microbiome derived from fecal of 22 moderate/severe TBI patients profiles with 18 healthy age-matched control subjects. The result found that bacteria in the *Ruminococcaceae* family were more abundant in chronic TBI in comparison to control profiles. However, Opeyemi et al^[30]^ observed that the microbial diversity is progressively diminished in the gut upon TBI, and bacteria with an origin traced to the *Ruminococcaceae* families were exhausted compared with control groups. In our study, a host-genetic-driven increase in *RuminococcaceaeUCG004* exhibited significant negative effects on FBI and TBI. Liu et al^[31]^ performed an observational study to analyze the 16S rRNA intestinal microbiome between the delayed neurocognitive recovery (dNCR) group and the non-dNCR group and found that the abundance of *Anaerofilum* was significantly enriched in the dNCR group. However, no *Anaerofilum* and BI have been reported to date. A recent study uncovered that the genus *Anaerofilum* was the only bacterial group identified that had a significant negative association with carcinoid syndrome (such as diarrhea).^[32]^ This study first revealed the protective role of *Anaerofilum* in FBI and TBI. Genus *LachnospiraceaeNC2004group* is an anaerobic bacteria belonging to the *Lachnospiraceae* family. Opeyemi et al^[30]^ reported that bacteria from *Lachnospiraceae* significantly decrease in the taxonomic composition of fecal samples post-TBI. These findings align with our study, which confirms the potential protective effects of *LachnospiraceaeNC2004group* in TBI and FBI. A possible explanation is that the *Lachnospiraceae* families entail dominant SCFA producers in mice and humans, because of their anti-inflammatory qualities and ability to affect immunological control, SCFAs may help lessen the impact of inflammation following BI.^[33]^ Additionally, research has shown that when drinking water is supplemented with SCFA, spatial learning is improved following TBI.^[30]^ Our results ascertained the distinct profiles of dominant taxa in FBI and TBI. These 2 share common characteristics despite also having differences. For example, genetically predicted higher relative abundances of *Coprococcus3* are significantly negatively associated with FBI, while positively associated with TBI. The difference in results is most likely due to 2 different BIs caused by different causes. Finally, we executed a reverse MR analysis with FBI/TBI-specific GM traits as the outcome and FBI/TBI as the exposure variables and found no significant reverse causal association of BI on FBI/TBI-specific GM. The reliability of the results is further improved. To sum up, further characterization of GM taxa, such as *RuminococcaceaeUCG004, Anaerofilum, LachnospiraceaeNC2004group, Escherichia.Shigella,* and *FamilyXIIIAD3011group* will avail a novel perspective for the development and progress of FBI/TBI and will aid in finding potential therapy for BI patients.

Several observational studies and animal experiments have found that immune cell infiltration (such as neutrophils, monocytes, microglia, astrocytes, T cells, and B cells) functions in the genesis and progression of BI,^[34]^ and rejuvenating the immune system supports brain repair after injury.^[35]^ Generally speaking, neutrophil numbers commence to diminish after BI, activated microglia in conjunction with astrocytes assemble at the injury site – isolating tissues that are injured from healthy tissues and allowing the restorative processes. Monocyte infiltration to the injury site is instrumental in the injured brain repair. The recruitment of T and B cells to the brain pathology locations at later time points can vary depending on the severity of the BI.^[36]^ In our study, we performed MR analysis to investigate the causal interrelationship between 731 immune phenotypes and TBI and FBI, respectively, to further elucidate the direct causal relationship between them. As a result, a total of 27 genetically predicted immunophenotypes and 39 genetically predicted immunophenotypes (such as B cell subtypes, T cell subtypes, Treg cells subtypes, DC subtypes, granulocyte, plasma cell, basophil) significantly affected FBI and TBI, respectively, using IVW method had the same direction as the findings of MR-Egger regression, WM, simple model and weight model. Notably, there are 9 shared immunophenotypes (only B cell and DC subtypes) that have a significant causal relationship with FBI and TBI. In contrast to well-known immune cell infiltrations (monocytes and T cells), our findings point out the causal functions of B cells and DC in BI and the related studies are very few so far. A B cell marker, OX33, has been affirmed for 4 to 6 days following experimental injury.^[37]^ Meissner et al^[38]^ ascertained that a chemokine linked to B cell chemotaxis (CCL20) is potentiated within 4 hours and continues out to 3 days postexperimental BI. There are scarce pieces of clinical studies that examine the effect of BI on B cell populations. Mrakovcic-Sutic et al^[39]^ delved into examining B cell (CD5+/CD19+) populations in 20 patients suffering from severe TBI by utilizing their peripheral blood and discovered that there were no substantial differences when contrasted with healthy controls. A study by 2 groups of scientists^[40,41]^ inferred that autoantibodies precise to CNS proteins were identified in BI patients’ serum, implying a broken tolerance. B cells initiate an immune response against antigens derived from the brain upon injury and a possible pathophysiological function for B cells throughout BI the recovery phase, which concurs with this research that propounds that various B cells (for instance IgD+ CD24− B cell %lymphocyte, BAFF-R on IgD− CD38− B cell, BAFF-R on IgD+ CD38− naive B cell, and BAFF-R on memory B cell) have a protective/negative role in brain damage. The involvement of DCs following BI has not been studied deeply in comparison to the other perspectives of immunity. DCs believed to function in neuroinflammation via processing antigens as well as presenting these antigens to T cells to start an immune response.^[42]^ DCs occur in limited numbers in the brain parenchyma in normal conditions, in particular, predominantly situated adjacent to cerebrospinal fluid, and continuously monitor the immune microenvironment of the CNS.^[43]^ In summary, our current analysis identifies new immunological directions in BI, where the host-genetic-driven change in B cell and DC infiltration significantly affect FBI and TBI. Further research is necessary in the future to provide new strategies for BI treatment.

Pieces of evidence have affirmed that GM functions in peripheral immune response and neuroinflammation following BI, modifying the lymphocyte populations.^[44]^ Numerous immune cells, like lymphocytes, participate in dynamic and complex immune responses in various BIs and maybe a central moderator in the GM and BI immunomodulation.^[44]^ Mediation analyses availed genetic evidence that various distinct immune cells mediate the causal impacts of the GM on BI. *Escherichia.Shigella* exerts its protective effects against FBI by increasing the abundance of infiltrating CD123 on CD62L+ plasmacytoid DC and CD123 on plasmacytoid DC. *FamilyXIIIAD3011group* increases the proportion of infiltrating CCR2 on myeloid DC to protect against TBI. In brief, DC may be an important bridge of immune regulation between GM and BI. In the intestines, the intestinal DCs exhibit a local immunomodulatory function. DCs that have intestinal tolerability and manifest greater levels of CD103, initiate differentiation of intestinal homing Treg cells in mesenteric lymph nodes (mLN).^[45]^ Following BI, the GM ultimately imparts a neuroprotective impact via inducing DCs in the mesenteric mLN to express remarkable levels of CD103 as well as inducing Treg cells to differentiate and produce IL-10 to suppress IL-17+γδT cells.^[46]^ These findings in conjunction with the findings of our study offer enlightenment into the causal relationship between the GM and the BI, specifically pertaining to the mediating effects of DC. There are limited shreds of literature connected with the function of DCs in BI via the brain–gut axis, which highlights the need for further investigation into the interaction between the BI, GM, and DC modulation in future studies.

The utilization of summary-level data gleaned from extensive GWASs and bidirectional MR design stood out as the current study’s strengths. The aforementioned design particularly averted bias as a result of reverse causation and confounding in order to derive accurate findings under the assumptions of MR. Moreover, consistent findings from numerous sensitivity analyses and validation of different data set results indicate the robustness of our findings. In spite of this remarkable sense, it is inevitable that limitations also existed in our study. Firstly, in the MiBioGen consortium, microbiome profile characterization employs 16S ribosomal RNA gene sequencing, that only permits resolution from the genus to phylum. More information about a particular species is availed by metagenomic sequencing. However, a prior MR analysis of the GM ascertained that the *P*-values were occasionally more remarkable for higher taxonomic units, encompassing genera or phyla, implying that species provided similar functions.^[47]^ Secondly, our findings failed to withstand a strict Bonferroni correction for multiple comparisons. However, as a hypothesis-driven approach, the MR study with some biological evidence was used to test epidemiologically established associations, irrespective of Bonferroni corrected *P* values. As a result of the causal relationship between GM, immune cells, and BI, we are taking the intersection of 2 BI GWAS datasets, which can be mutually verified, improving the reliability of the results. Thirdly, since more than 70 % of the study population constituted only European ancestry and variations exist in terms of host metabolism, lifestyle, and resident GM among people globally, there is a need to ascertain whether our findings have the likelihood of applying to a distinct ethnic group. Unequal distribution of genetic variants among distinct racial and ethnic groups can cause population stratification, and this can introduce potential bias in the outcomes of the study. There is a need to take caution when executing the generalizability of the study findings on other racial or ethnic groups. It is crucial for future studies to incorporate a more diverse population to improve the generalizability of the result. Fourthly, MR relies on the premise that a linear connection between exposure and outcome, however in reality, this relationship may be more intricate, encompassing nonlinear relationships as well as interactions with other genetic and environmental factors. For instance, a few genetic variants may exhibit a substantial impact on the outcome at heightened or diminished levels of exposure, or the influence of the exposure on the outcome might be mediated or moderated by other factors. Therefore, forthcoming MR studies should meticulously account for possible nonlinear and interaction impacts between the immunophenotypes, GM, and BI.

## 5. Conclusion

To our knowledge, this study is the first to comprehensively examine the causal relationships between GM, immune cells, and FBI/TBI. Our findings supported several potential causal links between several specific GM taxa with FBI/TBI. Additionally, CD123 on plasmacytoid DC and CCR2 on myeloid DC may have a key function in gut microbiota-host crosstalk in FBI and TBI, correspondingly. More research is paramount in the underlying mechanisms of GM in the occurrence of FBI/TBI for future development of probiotics to prevent and treat FBI/TBI.

## Acknowledgments

We thank Bullet Edits Limited for the linguistic editing and proofreading of the manuscript. We appreciate all the volunteers who participated in this study. We are grateful to the MiBioGen consortium and Open GWAS for providing GWAS summary statistics.

## Author contributions

**Conceptualization:** Lina Xian, Xiaochen Xu, Xiaoyan Deng.

**Data curation:** Lina Xian, Xiaochen Xu, Tongwu Guo, Zhen Chen, Xiaoyan Deng.

**Formal analysis:** Lina Xian, Xiaoyan Deng.

**Funding acquisition:** Lina Xian.

**Investigation:** Xiaoyan Deng.

**Methodology:** Xiaochen Xu, Tongwu Guo, Zhen Chen, Xiaoyan Deng.

**Project administration:** Xiaochen Xu, Xiaoyan Deng.

**Resources:** Xiaochen Xu, Yongmeng Mai, Xiaoyan Deng.

**Software:** Lina Xian, Yongmeng Mai, Xiaoyan Deng.

**Supervision:** Lina Xian, Zhen Chen, Xiaoyan Deng.

**Validation:** Lina Xian, Yongmeng Mai, Tongwu Guo, Xiaoyan Deng.

**Visualization:** Lina Xian, Xiaochen Xu, Yongmeng Mai, Tongwu Guo, Zhen Chen, Xiaoyan Deng.

**Writing – original draft:** Lina Xian, Xiaochen Xu, Xiaoyan Deng.

**Writing – review & editing:** Lina Xian, Xiaochen Xu, Yongmeng Mai, Tongwu Guo, Zhen Chen, Xiaoyan Deng.

## Supplementary Material


