# Supplementary material for: Dissecting causal relationships between gut microbiome, immune cells, and brain injury: A Mendelian randomization study
Source: Medicine (Baltimore). 2024 Sep 20;103(38):e39740. doi: 10.1097/MD.0000000000039740 (PMC11419422; doi:10.1097/MD.0000000000039740)
Supplement: Supplementary file 2 [file medi-103-e39740-s002.docx]

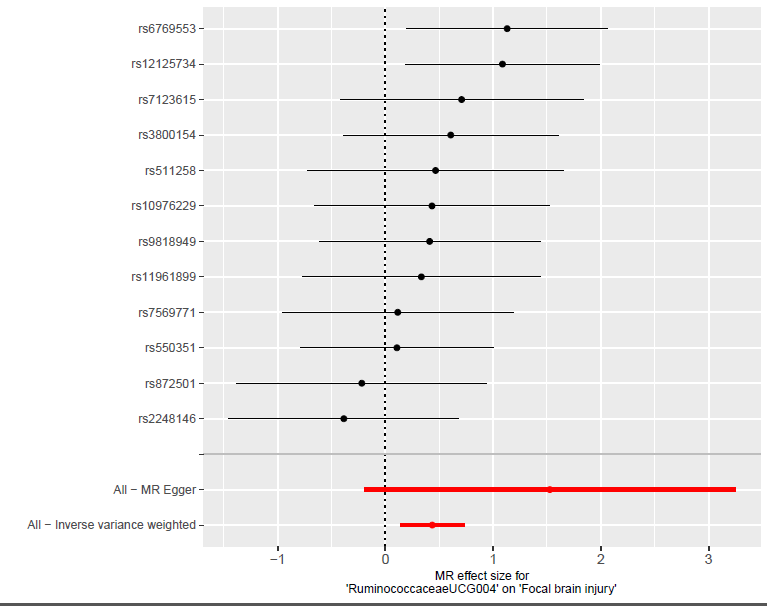


**Figure S1.** Forrest plot showing causal effect of genetically predicted significant 6 GM traits on FBI.


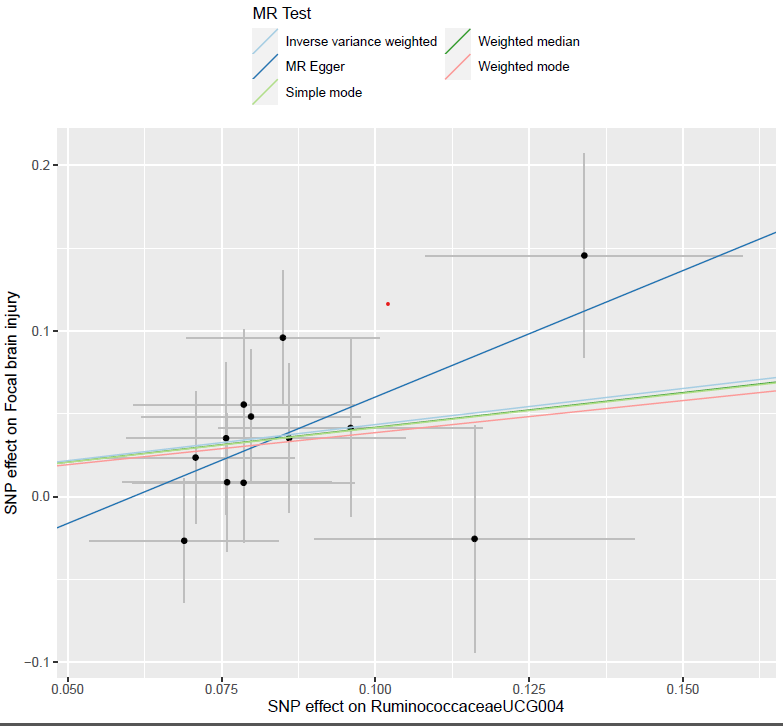


**Figure S2.** Scatter plot showing the association between significant 6 GM traits on FBI using five Mendelian randomization models. The x-axis indicates the SNP effect and standard error on GM traits for each of the SNPs, while the y-axis shows the SNP effect and standard error on FBI. The plot includes the regression line for MR-egger, weighted median, IVW, simple mode, and weighted mode.


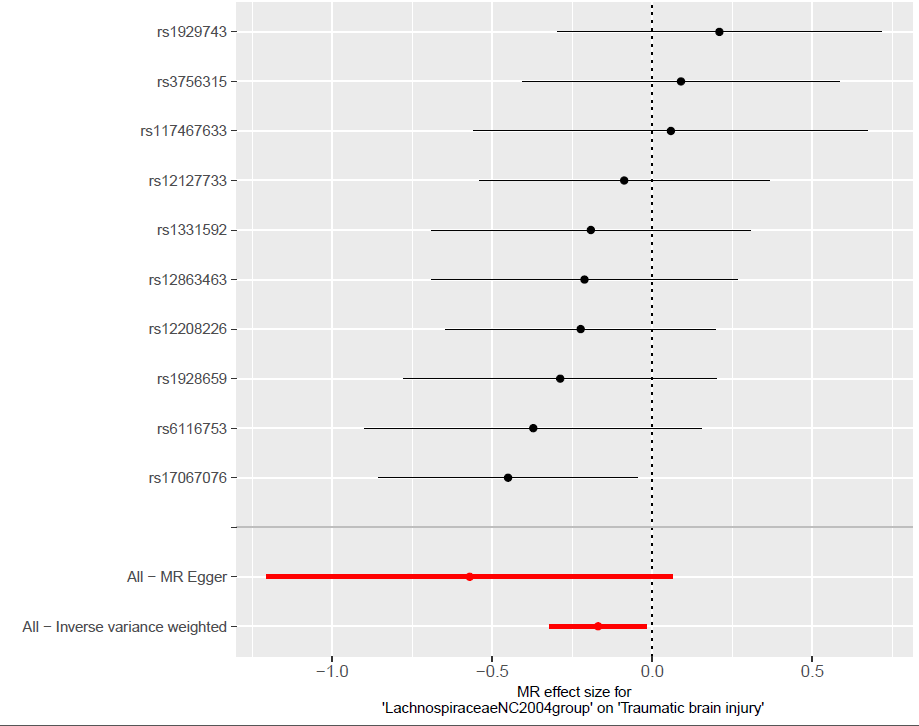


**Figure S3.** Forrest plot showing causal effect of genetically predicted significant 8 GM traits on TBI.


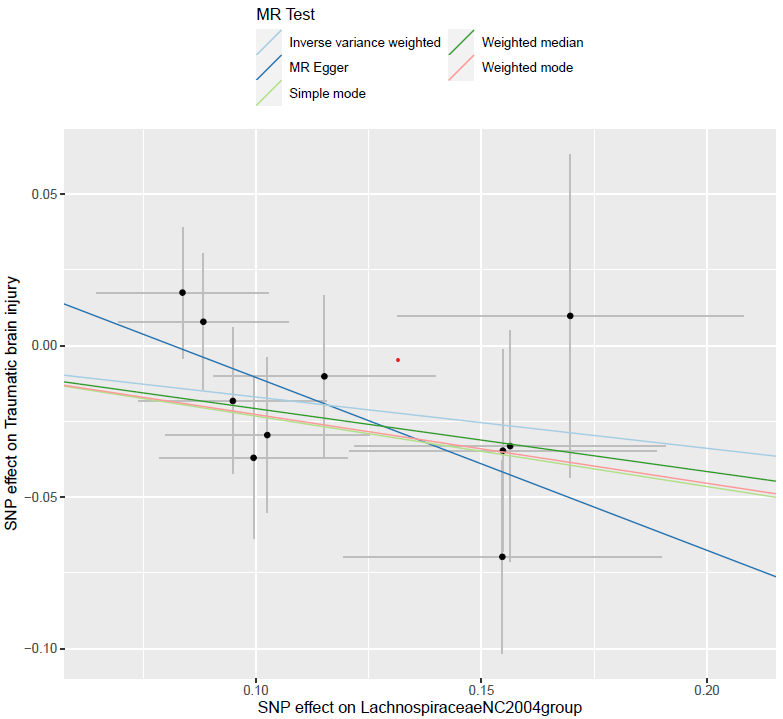


**Figure S4.** Scatter plot showing the association between significant 8 GM traits and TBI using five Mendelian randomization models. The x-axis indicates the SNP effect and standard error on immunological traits for each of the SNPs, while the y-axis shows the SNP effect and standard error on TBI. The plot includes the regression line for MR-egger, weighted median, IVW, simple mode, and weighted mode.


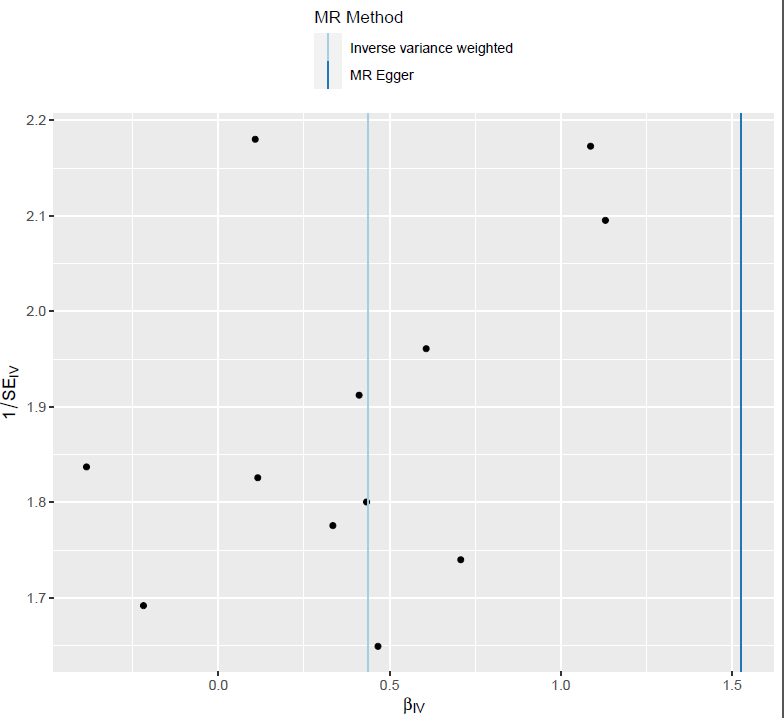


**Figure S5.** Funnel plot detecting heterogeneity of significant Mendelian randomization results using the MR-Egger regression and IVW methods (the causal effects of GM on FBI).


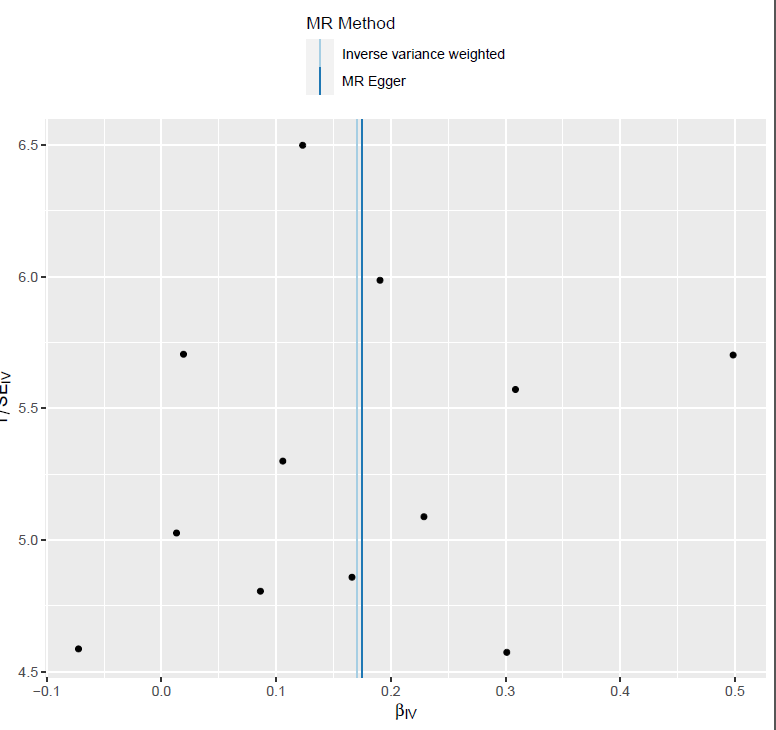


**Figure S6.** Funnel plot detecting heterogeneity of significant Mendelian randomization results using the MR-Egger regression and IVW methods (the causal effects of GM on TBI).


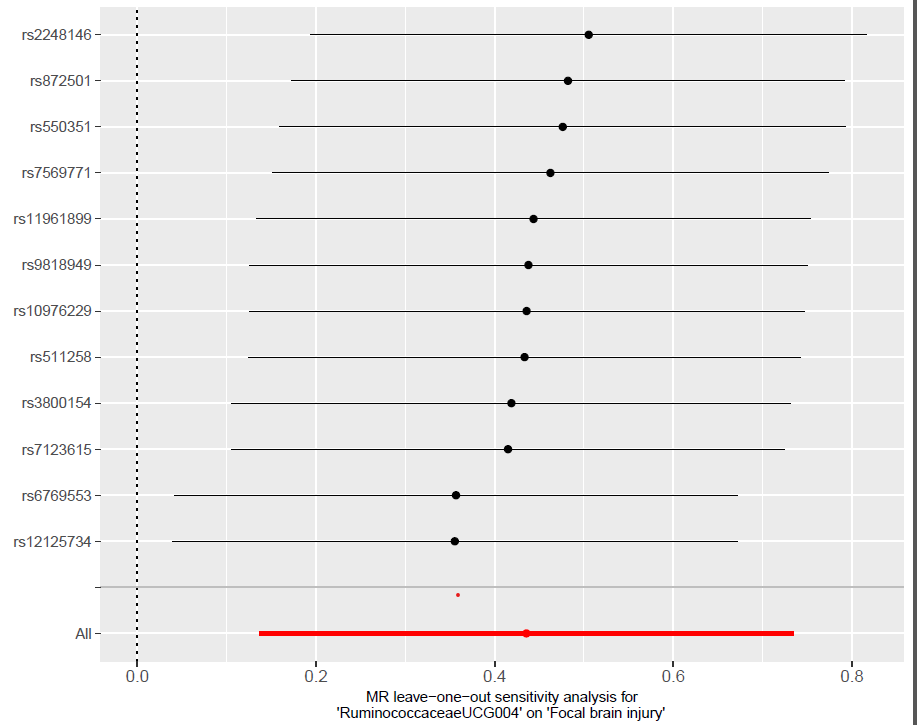


**Figure S7.** Presentation of the leave-one-out sensitivity analysis for the effect of 6 GM traits increasing SNPs on FBI risk in the context of MR. The dot and bar indicate the estimate and 95% CI when a specific SNP is removed.


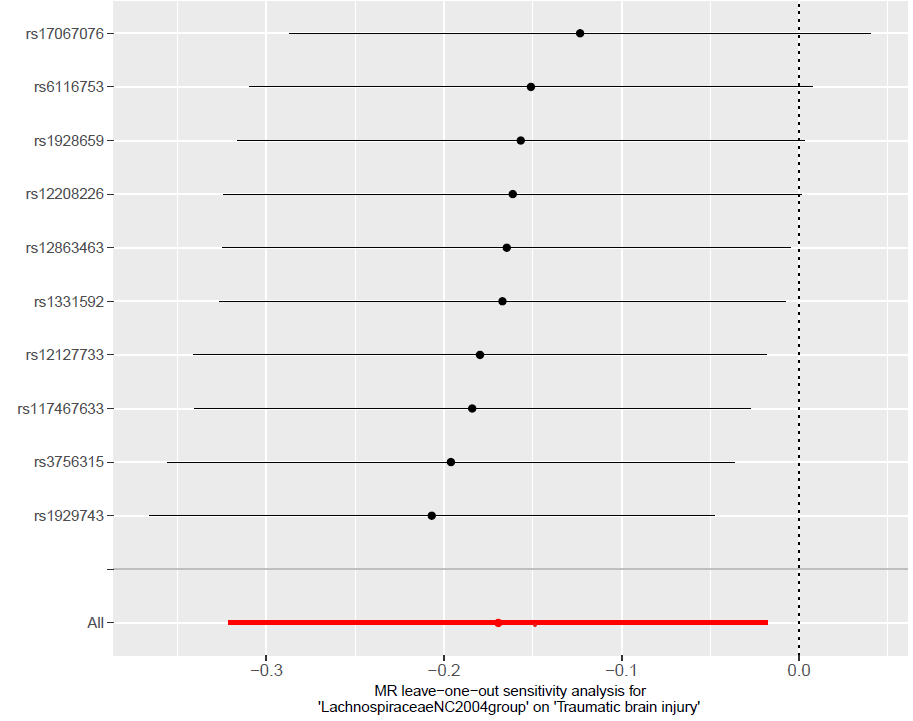


**Figure S8.** Presentation of the leave-one-out sensitivity analysis for the effect of 8 GM traits increasing SNPs on TBI risk in the context of MR. The dot and bar indicate the estimate and 95% CI when a specific SNP is removed.


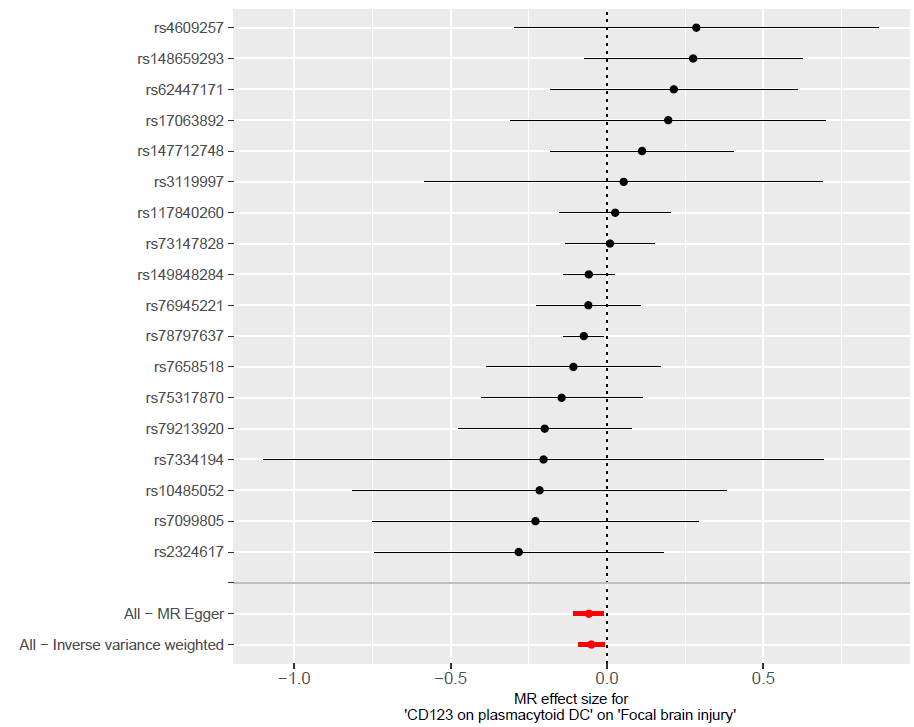


**Figure S9.** Forrest plot showing causal effect of genetically predicted significant 30 immunological traits on FBI.


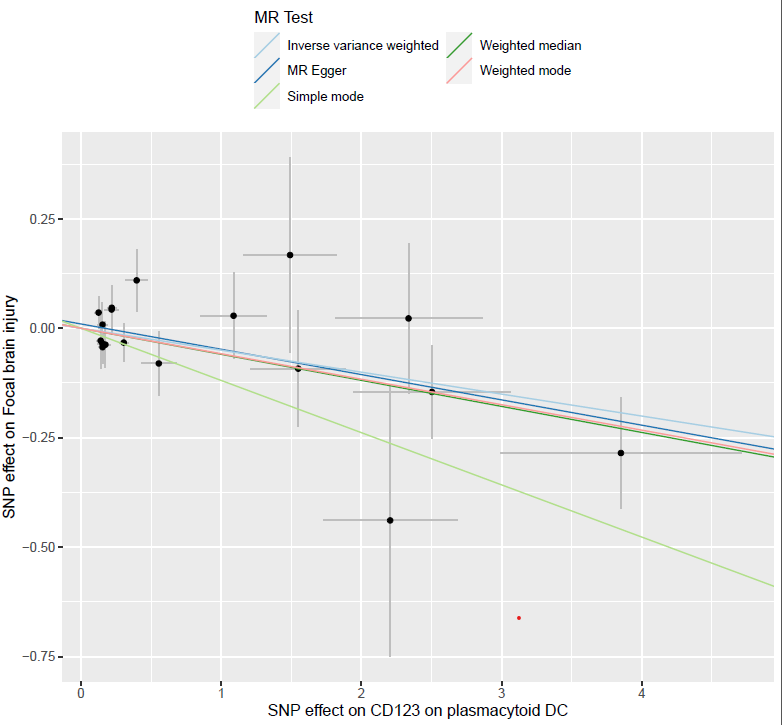


**Figure S10.** Scatter plot showing the association between significant 30 immunological traits and FBI using five Mendelian randomization models. The x-axis indicates the SNP effect and standard error on immunological traits for each of the SNPs, while the y-axis shows the SNP effect and standard error on FBI. The plot includes the regression line for MR-egger, weighted median, IVW, simple mode, and weighted mode.


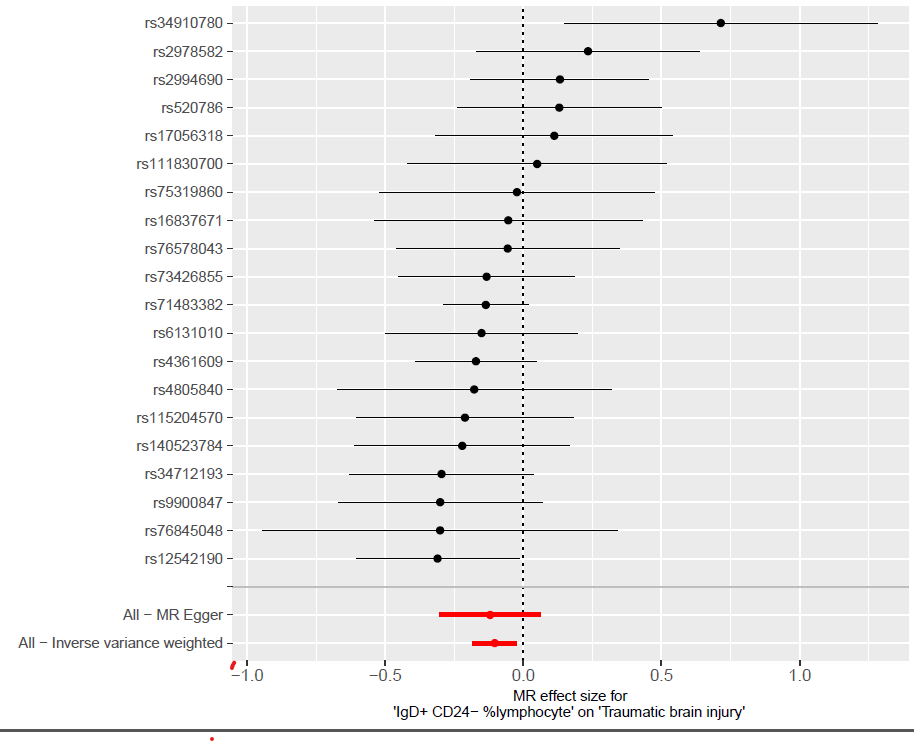


**Figure S11.** Forrest plot showing causal effect of genetically predicted significant 42 immunological traits on TBI.


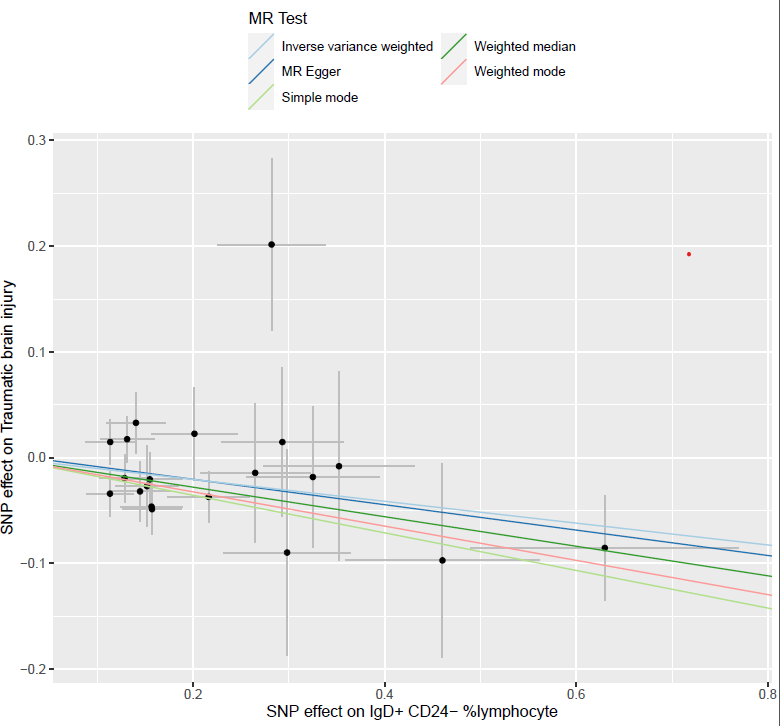


**Figure S12.** Scatter plot showing the association between significant 42 immunological traits and TBI using five Mendelian randomization models. The x-axis indicates the SNP effect and standard error on immunological traits for each of the SNPs, while the y-axis shows the SNP effect and standard error on TBI. The plot includes the regression line for MR-egger, weighted median, IVW, simple mode, and weighted mode.


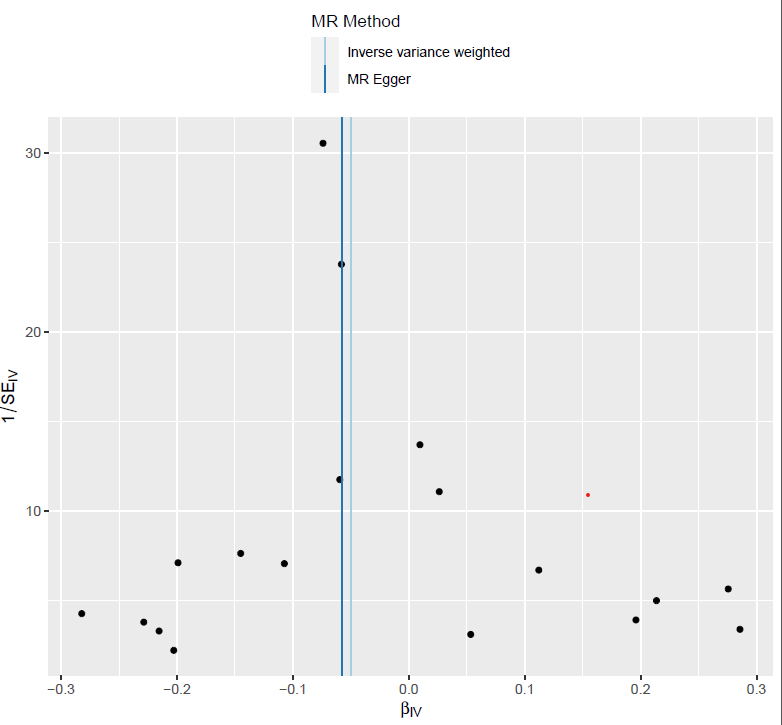


**Figure S13.** Funnel plot detecting heterogeneity of significant Mendelian randomization results using the MR-Egger regression and IVW methods (the causal effects of immune cells on FBI).


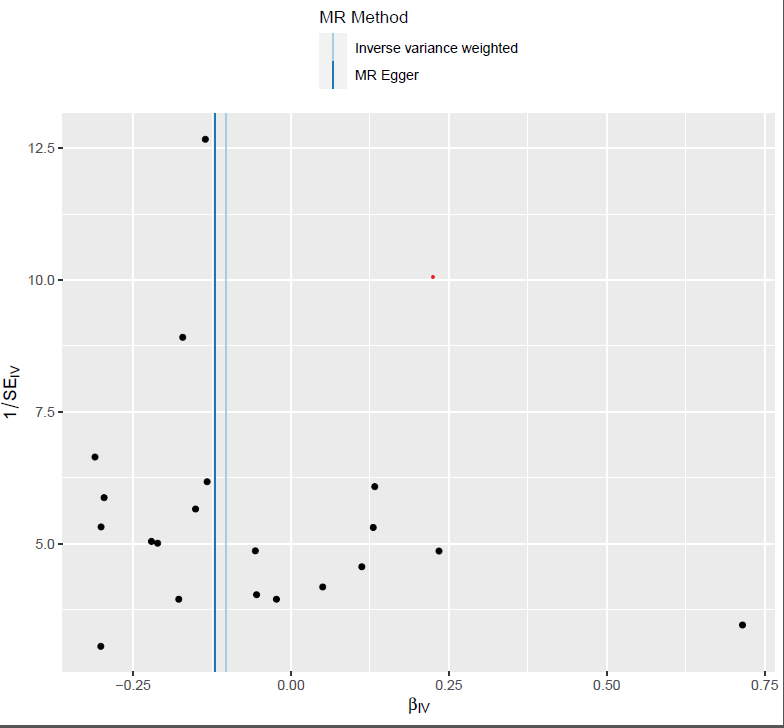


**Figure S14.** Funnel plot detecting heterogeneity of significant Mendelian randomization results using the MR-Egger regression and IVW methods (the causal effects of immune cells on TBI)


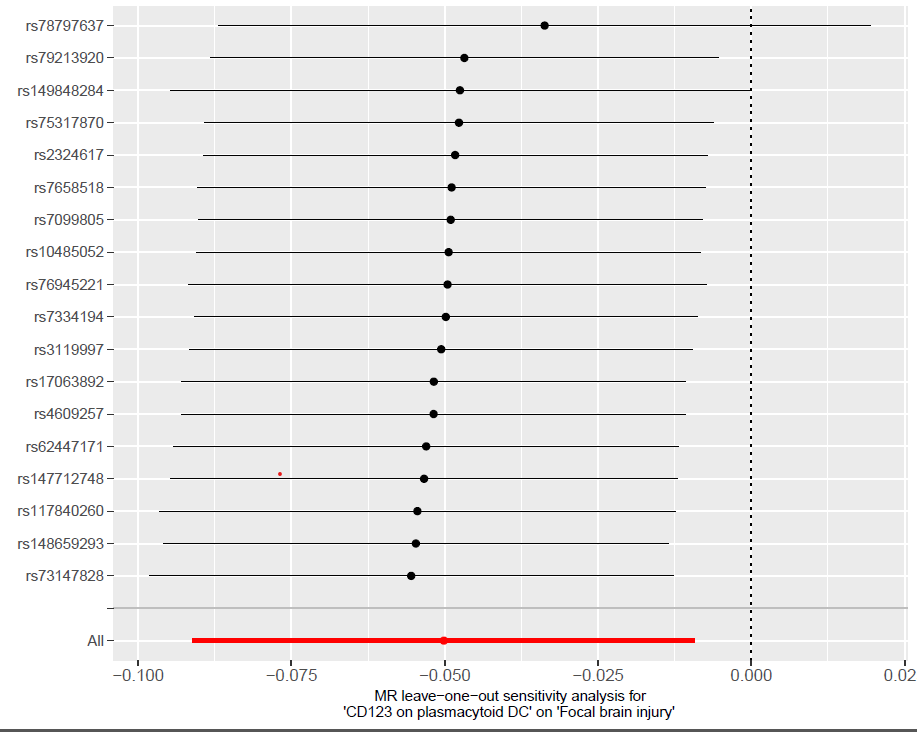


**Figure S15.** Presentation of the leave-one-out sensitivity analysis for the effect of 30 immunological traits increasing SNPs on FBI risk in the context of MR. The dot and bar indicate the estimate and 95% CI when a specific SNP is removed.


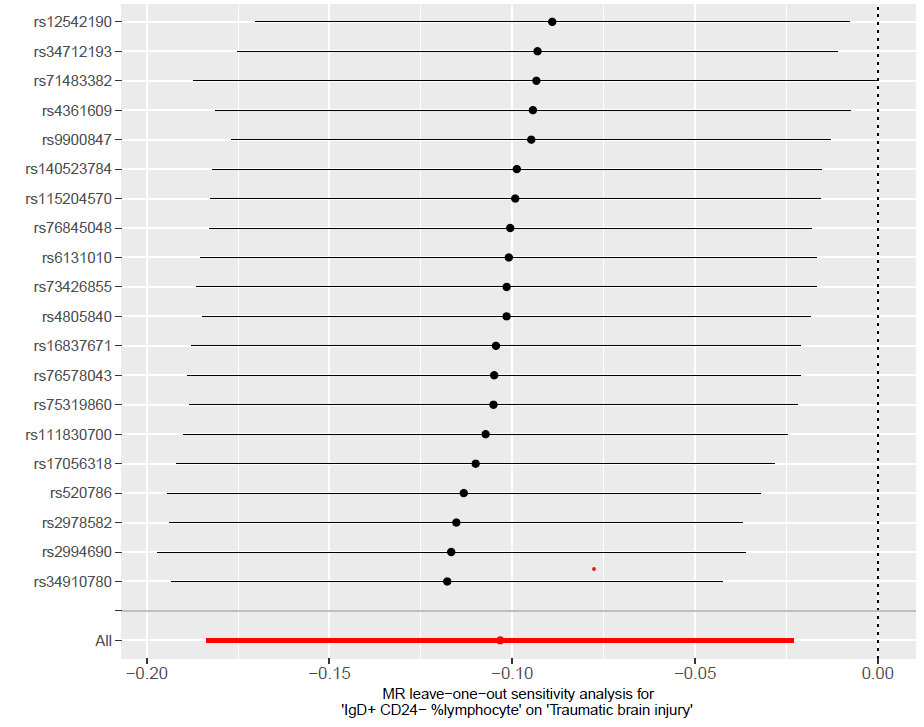


**Figure S16.** Presentation of the leave-one-out sensitivity analysis for the effect of 42 immunological traits increasing SNPs on TBI risk in the context of MR. The dot and bar indicate the estimate and 95% CI when a specific SNP is removed.
